# Three‐Segment Protein Labeling Using a Highly Efficient and Cysteine‐Less Split Intein Identified with Computational Prediction of Aggregation Properties

**DOI:** 10.1002/anie.202515821

**Published:** 2025-10-23

**Authors:** Christoph Humberg, Jonas Kröger, Shmuel Pietrokovski, Henning D. Mootz

**Affiliations:** ^1^ Department of Chemistry and Pharmacy, Institute of Biochemistry University of Münster Corrensstraße 36 48149 Münster Germany; ^2^ Department of Molecular Genetics Weizmann Institute of Science Rehovot 76100 Israel

**Keywords:** Click biology, Protein labeling, Protein ligation, Protein splicing, Thiol bioconjugation

## Abstract

Split inteins are versatile tools in protein engineering. We envisaged a new tandem protein *trans*‐splicing (PTS) scheme to assemble a protein from three segments, of which each can be individually treated with regard to its cysteine oxidation or chemical labeling status. However, only a single highly efficient cysteine‐less split intein has been reported so far. Split intein activities are currently not predictable and require time‐consuming biochemical characterizations. We aimed to accelerate the discovery of novel split inteins with high splicing efficiency by computational sequence analysis. Inspired by our previous finding that linked reduced splicing efficiency of characterized split intein fragments to soluble, β‐sheet‐rich amyloid‐like aggregates, we confirmed the inverse correlation between predicted aggregation propensity and splicing efficiency for new intein candidates by size‐exclusion chromatography and biochemical analysis. The LCGC14 intein emerged as a second available cysteine‐less split intein with virtually quantitative splicing efficiency, significantly expanding protein engineering opportunities independent of thiol chemistries or in oxidizing conditions. We exploit the orthogonality to the cysteine‐less CLm intein to assemble proteins from three selectively labeled segments, as demonstrated for a trimodular non‐ribosomal peptide synthetase (NRPS). The prediction of split intein efficiency from their sequence is a significant advancement to streamline future discovery processes.

## Graphical abstract

Tandem protein *trans*‐splicing (PTS) assembles a protein from three segments. Expanding this technology to individually treat cysteines in each segment, for example, for chemical labeling using thiol bioconjugation, the authors identify a second, highly efficient cysteine‐less split intein. Using computational, sequence‐based analysis of new split intein candidate genes, they streamline the split intein discovery process for the first time by eliminating candidates with a high aggregation propensity that have poor splicing yields.

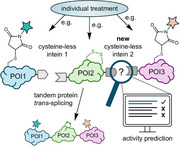

## Introduction

**Figure 1:**
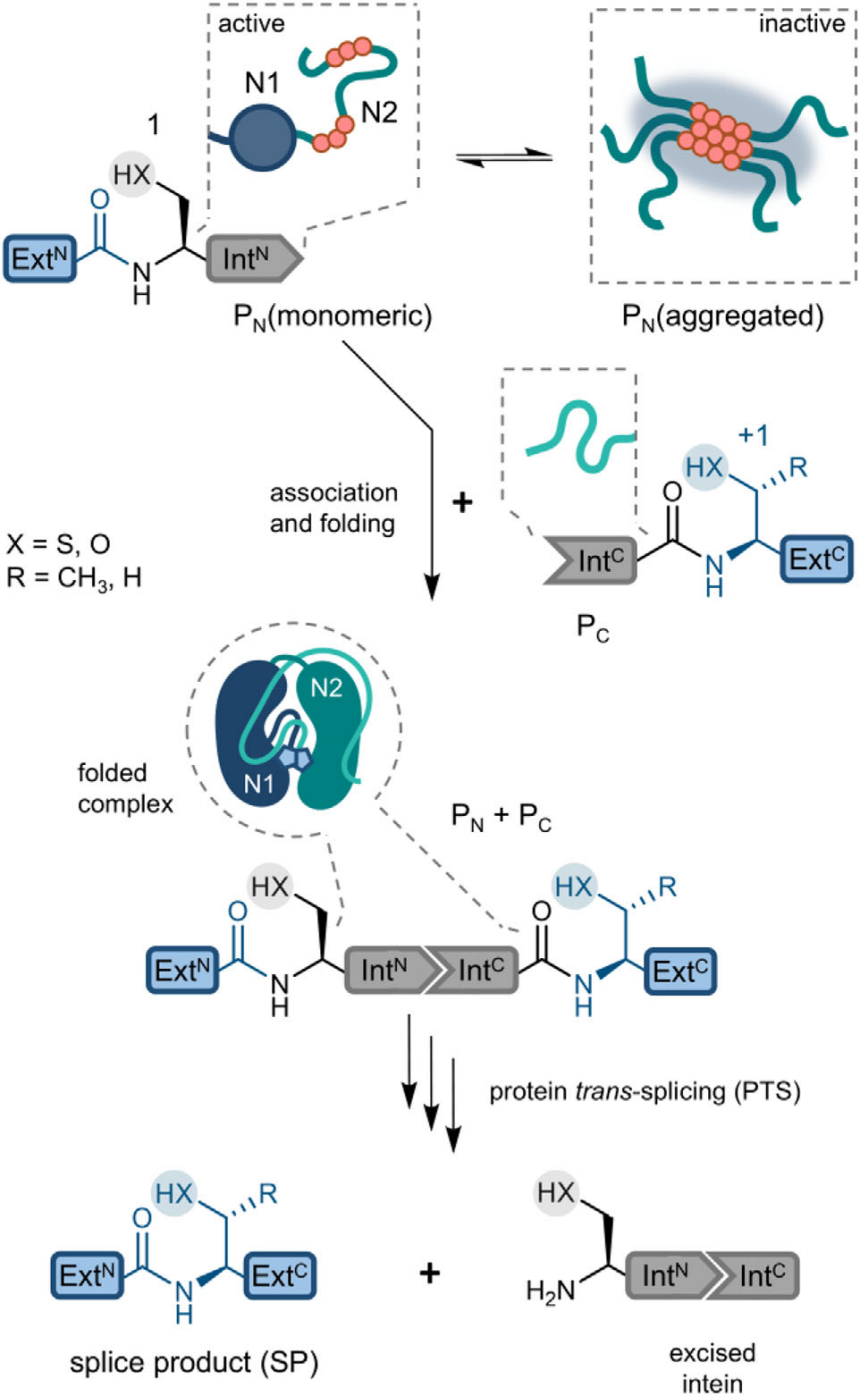
Split intein‐mediated PTS. Split inteins join their flanking sequences, the N‐ and C‐terminal exteins (Ext^N^ and Ext^C^), with a peptide bond. Cysteine‐less inteins operate with Ser1 and Ser+1/Thr+1 residues at the two splice junctions. The insets illustrate the folding states of typically split intein fragments. The Int^N^ fragment with its molten globule form of the N1 lobe and a disordered N2 lobe can be prone to the formation of soluble, yet inactive, aggregates. Red circles indicate aggregation‐prone regions.

**Figure 2:**
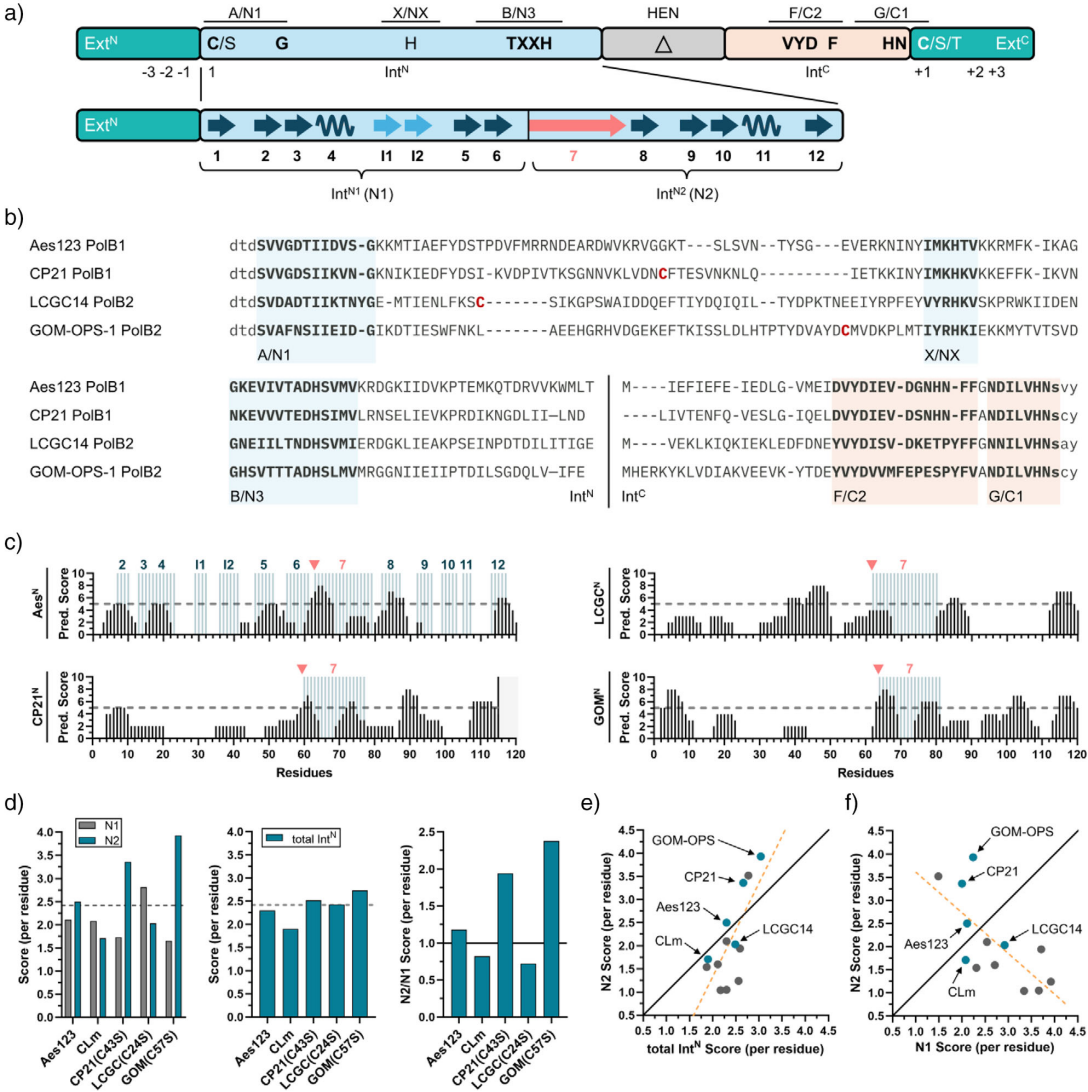
Sequence analysis of newly identified cysteine‐less split inteins. a) Shown are conserved sequence motifs of a full‐length *cis*‐intein (top) and secondary structure elements of the minimal intein horseshoe fold for the Int^N^ fragment of split inteins (bottom) as previously defined.^[^ ^19^ ^]^ β‐strands I1 and I2 are an optional insertion often found in cysteine‐less inteins.^[^ ^20^ ^]^ b) Multiple sequence alignment of the investigated cysteine‐less split inteins and the Aes split intein with highlighted conserved motifs. Non‐essential cysteines are shown in red. c) Consensus predictions of amyloidogenic pattern formation using AMYLPRED2^[^ ^21^ ^]^ of the Int^N^ parts. Secondary structure elements are highlighted for the Aes split intein based on its known structure.^[^ ^19^ ^]^ The red arrowhead illustrates the N1/N2 border. d) Aggregation scores of the indicated Int^N^ fragments for the N1 and N2 lobes (left panel), the entire Int^N^ (N1+N2) (middle panel), and calculated as the ratio of the N2/N1 scores (right panel). e), f) Plots of the N2 scores per residue against the overall Int^N^ score e) and against the N1 scores per residue f). This analysis includes some commonly used natively split inteins (as shown in Figure S2). Solid black lines indicate the theoretical line of a 1:1 positive correlation, and dashed red lines represent the linear regression fit of the experimental data.

Inteins are self‐processing proteins inserted within host proteins that catalyze post‐translational protein splicing.^[^ ^1^, ^2^ ^]^ This process involves cleavage of the peptide bonds flanking the intein domain and concomitant ligation of the flanking polypeptide sequences (exteins) through a new peptide bond to give the maturated host protein (see Figure 1).^[^ ^3^, ^4^, ^5^, ^6^ ^]^ The intein excision is virtually traceless, leaving only a single Cys, Ser, or Thr residue at the splice site. In split inteins, the intein domain is split into two fragments, with each of the N‐terminal and C‐terminal intein fragments (Int^N^ and Int^C^) residing on separate precursor proteins P_N_ and P_C_, respectively (Figure 2a).^[^ ^7^, ^8^ ^]^ Protein *trans*‐splicing (PTS) then occurs following high‐affinity intein fragment association and folding into the active intein complex through a series of acyl rearrangement reactions (Figure 1).^[^ ^9^, ^10^, ^11^ ^]^ These unique features make split inteins powerful protein engineering tools clearly distinguishable from other technologies, such as peptide ligase or SpyTag/SpyCatcher‐mediated protein conjugation.^[^ ^12^, ^13^, ^14^ ^]^ Split inteins have enabled a wide range of technologies useful in protein biochemistry and cellular biology, including protein reconstitution, site‐specific labeling, and conditional protein activation.^[^ ^15^, ^16^, ^17^, ^18^ ^]^ To this end, there is still great demand for novel or engineered split inteins with tailored properties for specific protein modification challenges.

However, split intein discovery is challenging, as they are sporadically present in a very wide range of protein hosts and organisms, and their activities cannot be predicted from their encoding DNA. The current discovery of useful split inteins proceeds through individual and time‐consuming biochemical characterization of each candidate. In particular, when investigated by reconstitution from their purified precursors, split inteins can differ with regard to their splicing yields, rates, and fragment affinity, for example.^[^ ^9^, ^22^, ^23^, ^24^, ^25^, ^26^ ^]^ Although split intein improvement by engineering approaches has achieved notable success in specific cases,^[^ ^27^, ^28^, ^29^, ^30^ ^]^ they remain resource‐ and time‐intensive. Additionally, inteins show varying tolerance to the sequence immediately flanking the splice junction.^[^ ^24^, ^28^, ^31^, ^32^, ^33^ ^]^ Favorable properties in these parameters are highly desirable for a powerful protein modification tool.

In naturally split inteins, the conserved β‐sheet‐rich horseshoe fold of the intein structure^[^ ^34^, ^35^ ^]^ (typically 130–150 aa) is split to give pairs of complementary Int^N^ and Int^C^ fragments with different sizes (Figure 2a). The most common split site corresponds to the typical insertion site of a homing endonuclease (HEN) found in many contiguous inteins, resulting in a larger Int^N^ fragment (∼100–120 aa) and a shorter Int^C^ fragment (∼35–40 aa).^[^ ^7^, ^36^ ^]^ In contrast, atypically split inteins feature a shorter Int^N^ (about 15–25 aa) and a correspondingly longer Int^C^.^[^ ^29^, ^37^ ^]^

We have recently reported that the incomplete splicing yields observed for the Int^N^ precursors of some split inteins in the PTS reaction can be explained by their formation of soluble, splice‐inactive aggregates (Figure 1).^[^ ^19^ ^]^ We proposed that the aggregate formation originates from the unique folding characteristics of the typically split intein precursors and their assembly pathway, which is well studied for some inteins, namely the *Ssp* DnaE,^[^ ^10^ ^]^ *Npu* DnaE^[^ ^11^ ^]^ and Aes123 PolB1 (Aes)^[^ ^19^ ^]^ split inteins. It was shown that the fragments of these inteins are largely in a disordered state that keeps them competent for folding with their complementary partner. Following an electrostatically driven association (capture), they undergo a disorder‐to‐order transition into the folded intein structure (collapse). More specifically, the Int^N^ fragment consists of two distinct lobes with different folding characteristics prior to intein assembly (Figure 1).^[^ ^11^, ^19^ ^]^ While the N‐terminal N1 lobe adopts a molten‐globule‐like structure, the C‐terminal N2 lobe is disordered due to a high content of charged amino acids (N1 and N2 lobes are not to be confused with conserved A/N1, B/N3, etc. block motifs,^[^ ^38^, ^39^ ^]^ see Figure 2a). Within the N2 lobe, regions with a high local excess of either positively or negatively charged residues, depending on the intein, exhibit charge complementarity to sequence regions in the equally disordered Int^C^ fragment.^[^ ^11^, ^25^ ^]^ This charge segregation between the two intein fragments facilitates the rapid electrostatic capture. The collapse into the thermodynamically stable fold is then driven by the high content of amino acids favoring β‐sheet formation, which are distributed along the intein sequence, including the disordered N2 lobe and the Int^C^ fragment. Importantly, the disordered nature of the polypeptide on the one hand and the high intrinsic propensity to promote stable folding into β‐sheets upon fragment association on the other hand represent an unusual dualism.^[^ ^19^ ^]^ It creates a challenge for the Int^N^ precursor to prevent misfolding in the absence of the Int^C^ partner by an alternative pathway leading into homomeric β‐sheet‐dominated aggregates, as demonstrated for the Aes and *Npu* DnaE split inteins.^[^ ^19^ ^]^

We wondered whether our insights on soluble aggregate formation of Int^N^ precursors could be leveraged to streamline the discovery process of novel split inteins that are highly active in terms of their splicing efficiency. Specifically, we were interested in identifying a new cysteine‐less split intein with favorable splicing efficiency. Cysteine‐less split inteins are a rare subclass of split inteins that exhibit only Ser or Thr residues at the catalytic 1 and +1 positions (Figure 1).^[^ ^40^ ^]^ Importantly, unlike the more prevalent cysteine‐dependent split inteins with Cys residues at only one of these positions that have enabled selective Cys labeling in the other precursor protein,^[^ ^41^, ^42^, ^43^, ^44^ ^]^ cysteine‐less split inteins are insensitive to oxidative conditions and do not require reducing agents for efficient splicing.^[^ ^45^ ^]^ Furthermore, they are fully compatible with thiol‐based bioconjugation reactions.

These features of cysteine‐less split inteins increase the scope of possible extein proteins and labeling schemes for protein modification.^[^ ^19^, ^41^, ^45^, ^46^, ^47^ ^]^ To this end, we envisaged that tandem PTS^[^ ^9^, ^48^, ^49^, ^50^, ^51^ ^]^ with two orthogonal cysteine‐less split inteins would enable powerful novel combinations of PTS technologies with other protein chemistries. However, the only two efficiently splicing cysteine‐less split inteins, the engineered CLm and CL inteins, are both derived from the same aggregation‐prone and incompletely splicing Aes split intein and thus lack orthogonality, as we show in this study. The ultra‐fast CLm split intein was engineered through the introduction of aggregation‐suppressing mutations in the Aes^N^ fragment,^[^ ^19^ ^]^ while the moderately fast CL intein was obtained by shifting the split site closer to the N terminus.^[^ ^45^ ^]^ A second highly efficient and orthogonal cysteine‐less split intein is still missing.

In this work, we selected previously uncharacterized cysteine‐less split intein candidates from publicly available databases. By sequence‐based bioinformatic analysis we predicted the propensity of their Int^N^ fragments to form inactive β‐sheet‐dependent aggregates. Following biochemical characterization, we identified the LCGC14 PolB2 (LCGC14) split intein as the second available cysteine‐less split intein that is highly efficient in PTS. We exploit its orthogonality to the CLm intein by a new tandem PTS scheme in which each of the three linked protein segments is individually addressable with regard to its chemical cysteine modification. To the best of our knowledge, our work shows for the first time that an important activity trait of uncharacterized inteins can be predicted from the sequence, simplifying the discovery of well‐active new split inteins. The novel LCGC14 split intein expands the toolbox of cysteine‐less split inteins.

## Results and Discussion

### Strategy to Identify High Splicing Efficiency of Novel Cysteine‐Less Split Inteins

We aimed to streamline the identification process of highly efficient split inteins. Identifying aggregation‐prone split intein candidates prior to biochemical characterizations would significantly reduce the experimental effort in attempts to find promising novel split inteins. Here, we were specifically interested in identifying a novel, naturally occurring, cysteine‐less split intein with high splicing efficiency to expand the intein toolbox.

### Identification of Three Novel Cysteine‐Less Split Intein Candidates

We identified three novel and previously uncharacterized cysteine‐less split intein candidates in PolB‐type DNA polymerases from T4‐like bacteriophages of *Campylobacter* phage CP21 and from metagenomic datasets of marine sediments. The CP21 PolB1, LCGC14 PolB2, and GOM‐OPS‐1 PolB2 split inteins exhibit sequence identities of 52.4%, 35.0%, and 31.7%, respectively, relative to the wild‐type Aes intein.^[^ ^45^ ^]^ These typically split inteins exhibit predicted Int^N^ fragment lengths of 120 amino acids (aa) for LCGC14 (LCGC^N^ hereafter) and GOM‐OPS‐1 (GOM^N^) inteins and 115 aa for CP21 (CP21^N^). The lengths of the complementary Int^C^ fragments are 42 aa for LCGC^C^, 46 aa for GOM^C^, and 39 aa for CP21^C^. Figure 2b shows a multiple sequence alignment of these 4 split inteins. All three new split inteins utilize serine at positions 1 and +1. However, each intein contains a cysteine at a non‐conserved position (Figure 2b). The recently discovered NX motif with a histidine, highly conserved only in cysteine‐independent inteins,^[^ ^20^ ^]^ was found in all three split inteins.

### Sequence‐Based Prediction of the Aggregation Propensity Reveals One Promising Split Intein Candidate

We previously showed that incomplete splicing of Int^N^ precursors can be caused by their formation of soluble, β‐sheet‐based aggregates that are inactive in splicing.^[^ ^19^ ^]^ For the Aes split intein, we showed that the disordered N2 lobe of the Int^N^ precursor drives the aggregation. We further showed that the introduction of single amino acid mutations designed to reduce the predicted aggregation propensity within regions of the N2 lobe indeed led to a complete abolishment of aggregate formation in the engineered CLm^N^ precursor.^[^ ^19^ ^]^ Based on these insights, we here asked whether the aggregation propensity of novel split inteins could be computationally predicted to sort out candidates with unfavorable aggregation properties.

We used multiple sequence alignments and structural prediction (AlphaFold 3)^[^ ^52^ ^]^ of the three new split inteins to identify the region of each Int^N^ fragment that corresponds to the disordered N2 lobe of the Aes^N^ fragment (Figure S1). In the folded structure of the Aes intein^[^ ^19^ ^]^ this region represents the C‐terminal of the two nearly symmetrical halves^[^ ^53^ ^]^ of the Aes^N^ fragment^[^ ^19^ ^]^ plus the remaining upstream amino acids belonging to the long β‐strand β7 connecting the N‐ and C‐terminal halves (highlighted in Figure 2a). β7 interacts with β13 from the Int^C^ fragment, and these sequence regions account for most of the residues forming clusters with opposite charges that are thought to account for the electrostatically driven initial contact in the association of the disordered Int^N^ and Int^C^ precursors.

We then employed the web tool AMYLPRED2 to analyze the sequence‐based aggregation propensity of all three new Int^N^ sequences (Figure 2c).^[^ ^21^ ^]^ For these analyses, we changed the single non‐conserved cysteine in each Int^N^ fragment to serine, as we were interested in the fully cysteine‐less split inteins. We obtained clearly different values for the calculated average aggregation scores of the N2 lobes (Figure 2d, left panel). According to this analysis, both the CP21^N^ and the GOM^N^ fragments should be more aggregation‐prone than the Aes^N^ fragment, which is shown for comparison. In contrast, the LCGC^N^ fragment exhibited a lower score for N2 than the aggregation‐prone Aes^N^ fragment, close to the score calculated for the engineered CLm^N^ fragment (Figure 2d). This analysis suggested that the LCGC^N^ fragment should exhibit a low tendency to form aggregates.

Given the significant differences seen for the average aggregation scores of the N2 lobes among the different split inteins (Figures 2d and S2), we wondered how these would influence the aggregation score of the entire Int^N^ fragments. Notably, as the aggregation score is primarily driven by hydrophobic residues, this value also reflects the order‐promoting features of a sequence and ultimately will be related to the stable folding with the Int^C^ partner into the intein structure rich in β‐sheets. Maybe not surprisingly, the plot in Figure 2e shows that the N2 score of various well‐known native split inteins exhibits a positive correlation with their overall Int^N^ aggregation score (*ρ* = 0.72 (*p* = 0.0051)). Nevertheless, the overall Int^N^ aggregation scores appeared more comparable across different inteins than the N2 scores of the same fragments (Figure 2d, middle panel; Figure S2a,b). The explanation for this discrepancy can be found in the more significant differences between the aggregation scores for the N1 and N2 lobes of each Int^N^ fragment (Figure 2d, left panel; Figure S2b). Indeed, plotting the N2 score against the N1 score revealed a negative correlation *ρ* = −0.71 (*p* = 0.0069), indicating that a decrease in the N2 score correlates with an increase in the N1 score and vice versa (Figure 2f). We explain this relationship by an evolutionary pressure on split inteins against excessive aggregation propensity in the N2 lobe, as such aggregation would lower the splicing efficiency. To compensate for the reduced order‐promoting properties of the N2 lobe, which may ultimately compromise the split intein's capability for proper folding into the active intein structure, such order‐promoting features might in turn have accumulated in the N1 lobe. However, despite the concomitantly increased aggregation propensity in the N1 lobe, this does not lead to aggregate formation in a comparable fashion as observed for the disordered N2 lobe, likely due to the more molten globule‐like structural properties of the N1 lobe.

Taken together, we propose that the ratio of the N2 and N1 aggregation scores represents a more absolute metric to predict the propensity of an Int^N^ precursor to form inactive aggregates, compared to relying solely on the relative N2 scores. Indeed, many efficiently splicing split inteins show an N2/N1 ratio <1, while a ratio >1 is observed for split inteins known to be inefficient in splicing, like the native Aes intein or artificially split inteins derived from contiguous inteins (Figure S2c–f). Similarly, the low N2/N1 ratio of 0.72 calculated for the LGCG^N^(C24S) fragment suggests a high likelihood of this precursor being efficient in splicing, while the high ratios observed for the CP21^N^(C43S) (1.94) and GOM^N^(C57S) (2.38) fragments would predict to correlate with a high aggregation propensity (Figure 2d, right panel).

### Experimentally Determined Aggregate Formation of Int^N^ Precursors Correlates with Computational Prediction

We next experimentally tested if the results from the sequence‐based aggregation prediction analysis correlated with the ratio of monomeric and aggregated Int^N^ precursor proteins of the three new inteins.

We produced the Int^N^ precursors as recombinant fusion proteins MBP‐Int^N^‐His_6_ in *Escherichia coli* using maltose binding protein (MBP) as the model extein sequence with three native residues immediately flanking the intein. The single cysteine at non‐conserved positions found in each Int^N^ fragment was removed by site‐directed mutagenesis to serine. The encoded proteins MBP‐CP21^N^(C43S)‐H_6_ (**1P**), MBP‐LCGC^N^(C24S)‐H_6_ (**2P**), and MBP‐GOM^N^(C57S)‐H_6_ (**3P**) were expressed at 18 °C, purified by Ni‐NTA affinity chromatography, and dialyzed overnight at 4 °C to remove the imidazole.

**Figure 3:**
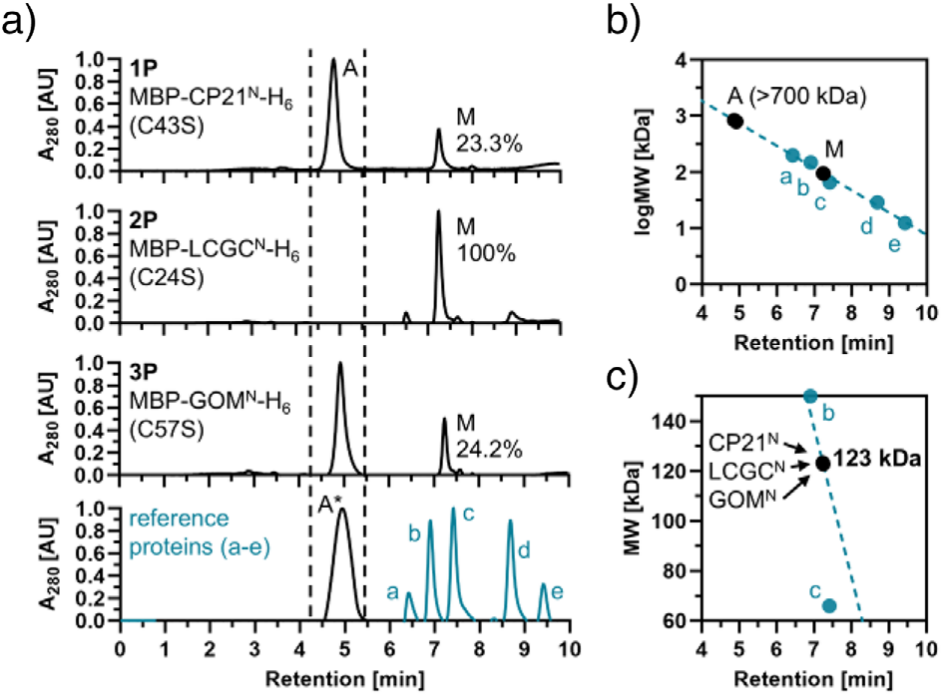
SEC analysis of purified Int^N^ precursor proteins (P_N_). a) SEC profiles of the investigated constructs **1P**, **2P**, and **3P**, each diluted to 10 µM and pre‐equilibrated for 24 h, as well as a set of globular protein markers used as size standards. The aggregated form of MBP‐CLm^N^‐H_6_ (**10P**), eluting at the void volume of the column,^[^ ^19^ ^]^ was used as a reference for an aggregated species (A*). b) Linear calibration plot of log MW versus retention time to estimate the apparent MWs of the aggregated and monomeric species of **1P**, **2P**, and **3P**. c) Linear calibration plot of MW versus retention time to calculate the apparent MW of the monomeric species. A = aggregated species; M = monomeric species; MW = molecular weight.

We then used size exclusion chromatography (SEC) to analyze different folding species. Following dilution of each protein to 10 µM and an incubation for 24 h at 15 °C to allow for a potential concentration‐dependent equilibration of the monomer to aggregate ratio,^[^ ^19^ ^]^ SEC revealed high proportions of soluble high molecular weight (MW) aggregates for **1P** and **3P** (77% and 76%, respectively), while the LCGC^N^(C24S) precursor **2P** showed no detectable aggregates (Figure 3a). The aggregates eluted at the void volume of the analytical SEC column (AdvanceBio SEC 200 Å). By using MW standards, we calculated an expected MW of at least 700 kDa for the aggregated species (Figure 3b). The retention times observed for the obviously monomeric species exceeded the calculated monomer MWs (57–58 kDa), which is not surprising considering they exhibit a partially unfolded structure (Figure 3c).

In control experiments we analyzed constructs MBP‐CP21^N^‐H_6_ (**4P**), MBP‐LCGC^N^‐H_6_ (**5P**), and MBP‐GOM^N^‐H_6_ (**6P**) with the native Int^N^ fragments containing the non‐essential single cysteines. Only slight deviations were predicted for their aggregation propensities compared to the cysteine‐less mutant sequences (Figures S2 and S3a,b). SEC experiments revealed qualitatively similar results with a non‐aggregated LCGC^N^ precursor and slightly higher percentages of aggregating CP21^N^ and GOM^N^ precursors (Figure S3c; 90% and 78% aggregated species for **4P** and **6P**, respectively). This analysis also revealed partial dimer formation for **5P** (27%); however, obviously not via an aggregation‐dependent mechanism but likely caused by intramolecular disulfide formation, because the dimeric species disappeared in favor of the monomeric species upon treatment with TCEP as a reducing agent (Figure S3c).

### The LCGC14 Split Intein Splices Rapidly and Virtually Quantitatively

**Figure 4:**
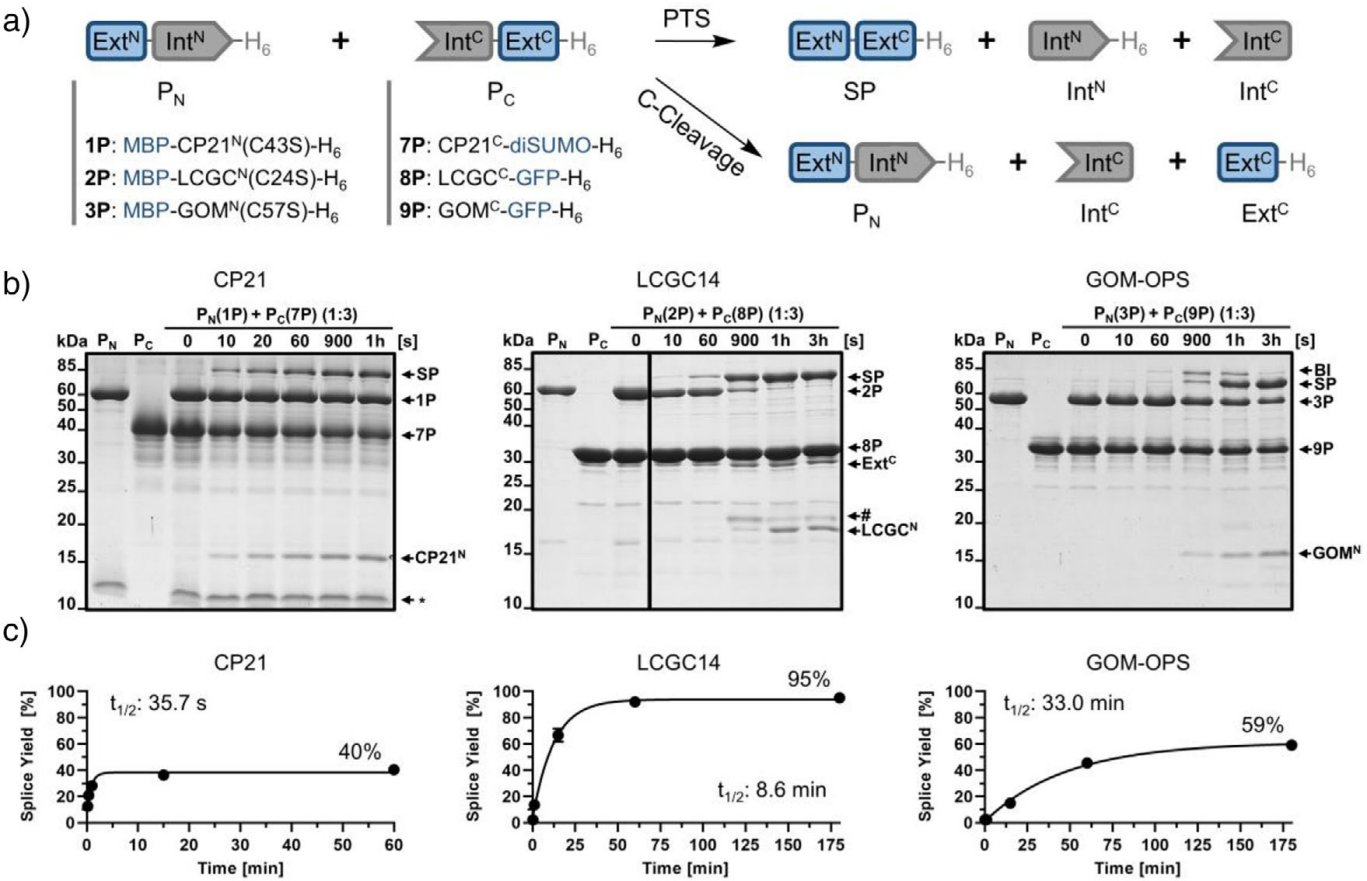
PTS activity of the new cysteine‐less split inteins. a) Scheme of the PTS reaction with C‐cleavage as a potential side reaction. b) Analysis of the PTS reactions between each pair of P_N_ (10 µM) and P_C_ (30 µM), performed at 37 °C and in the absence of reducing agents. Shown are Coomassie‐stained SDS‐PAGE gels. These experiments were repeated two times. Note that the band marked with a hash symbol (#) appears to be identical with the LCGC^N^ fragment according to an MS analysis (Figures S4 and S5) but shows an altered electrophoretic mobility for unknown reasons. c) Plots of splice product formation over time determined by densitometric analysis of data as shown in b) and fitted to a one‐phase exponential equation. Note that the MWs of the Int^C^ fragments excised during the PTS reactions are too small to be visible on these gels. MWs are shown in Table S1. (*) denotes a protein contamination. Uncropped SDS‐PAGE images are shown in Figure S12.

We next asked whether the highly monomeric nature of the Int^N^ precursors correlated with high splicing efficiency. We recombinantly produced and purified the three Int^C^ fragments with either super‐folder green fluorescent protein (sfGFP) or a linear dimer of small ubiquitin‐related modifier 2 (diSUMO) as model extein sequences to give CP21^C^‐diSUMO‐H_6_ (**7P**), LCGC^C^‐sfGFP‐H_6_ (**8P**), and GOM^C^‐sfGFP‐H_6_ (**9P**). Again, we used three native extein residues at the splice junctions, while the cysteine at position +2 in the GOM‐OPS‐1 intein was replaced by alanine. To trigger the PTS reactions, the Int^C^ precursor proteins (P_C_) from each pair were mixed with their P_N_ counterparts **1P**, **2P**, and **3P**, respectively, at 37 °C and without the addition of reducing agents. P_C_ to P_N_ ratios of 3:1 (10 µM P_N_ and 30 µM P_C_) were used to simulate a pseudo‐first‐order reaction (Figure 4a). For the CP21 split intein, we observed an ultra‐fast reaction rate of 19.4 ± 5.1 × 10^−3^ s^−1^ (*t*_1/2_: 35.7 s) but only incomplete splice product formation of the P_N_ (ca. 40%) (Figure 4b,c). Likewise, we observed only partial splicing for the GOM‐OPS‐1 split intein of about 59% efficiency relative to the P_N_, and with a much slower reaction rate of 0.33 ± 0.02 x10^−3^ s^−1^ (*t*_1/2_: 33.0 min). No further turnover was observed for both inteins after prolonged incubation. In contrast, for the LCGC14 split intein, we observed nearly quantitative splicing (95%; Figure 4b,c, middle panels). The reaction proceeded at a rate of 1.3 ± 0.1 × 10^−3^ s^−1^ (*t*_1/2_: 8.6 min), ranking the LCGC14 split intein as a relatively fast intein. While the P_N_ was completely consumed into the splice product, the excess P_C_ also resulted in some C‐cleavage of the GFP extein (<9%). LC–MS analysis confirmed the complete consumption of P_N_ and correct formation of the splice product MBP‐sfGFP‐H_6_ (Figures S4 and S5). Control experiments using a P_N_ to P_C_ ratio of 3:1 showed that each of the C‐terminal precursors (P_C_) could be completely consumed in the PTS reaction (Figure S6). Together these results showed that aggregation of the Int^N^ precursors (P_N_) is the underlying cause for the incomplete splicing of the CP21 and GOM‐OPS‐1 split inteins, while the monomeric LCGC^N^ was capable of virtually quantitative splicing.

In further control reactions we also investigated the PTS reactions of the three split inteins with their native Int^N^ precursors **4P**, **5P**, and **6P**, respectively, each containing a single, non‐conserved cysteine. Consistent with the higher levels of aggregate formation observed for MBP‐CP21^N^‐H_6_ (**4P**) and MBP‐GOM^N^‐H_6_ (**6P**) compared to the fully cysteine‐less variants, we observed further reduced splicing efficiencies for these split intein pairs (Figure S7). Likewise, compared to its fully cysteine‐less variant **2P**, the construct MBP‐LCGC^N^‐H_6_ (**4P**) showed reduced splicing efficiency (82%) with its Int^C^ precursor **8P**. However, in this case the lower yield could be explained by the aforementioned partial disulfide‐dependent dimer formation of **5P** (Figure S3c), as it could be alleviated to virtually complete splicing by the addition of a reducing agent (1 mM TCEP; Figure S7d). The reaction rates in PTS were largely unaffected by the cysteine removal (Table S2).

### The LCGC14 Split Intein is Orthogonal to the CLm Split Intein, Enabling New Schemes in Thiol‐Independent Protein Engineering

**Figure 5:**
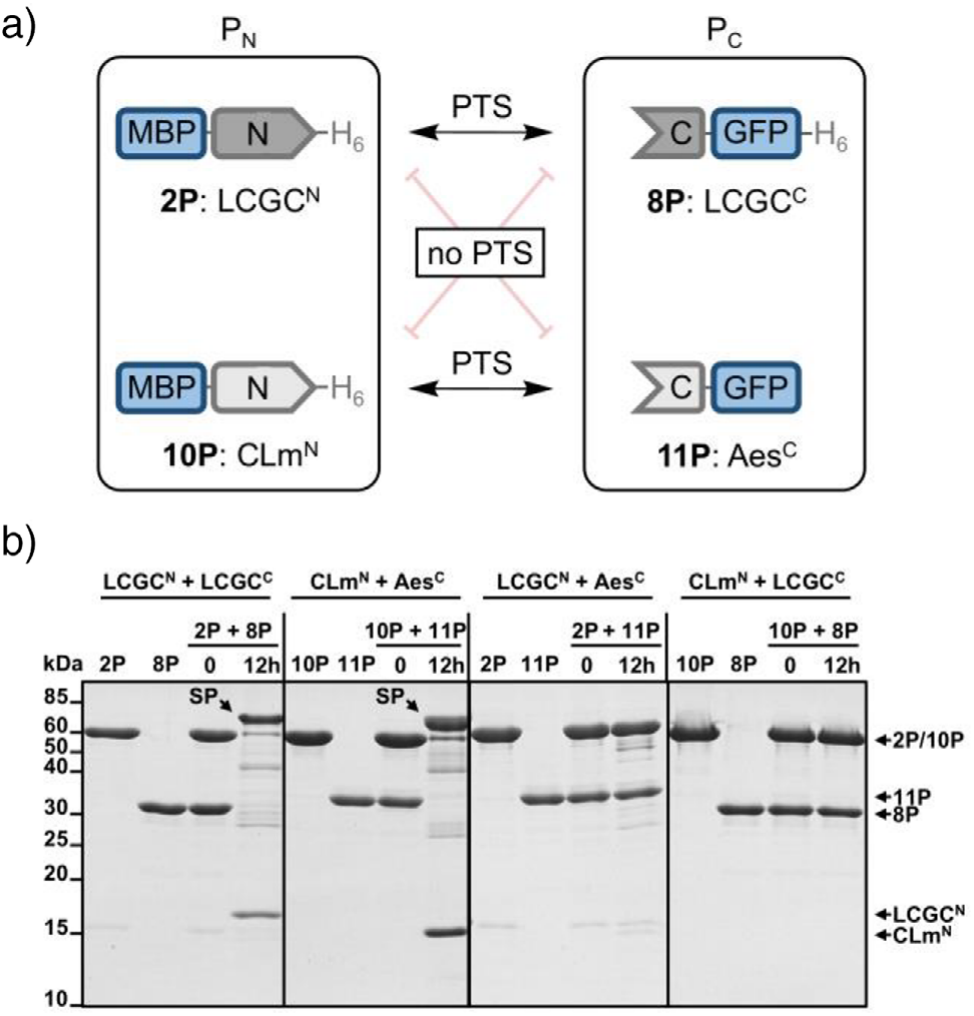
Orthogonality of the LCGC14 split intein to the cysteine‐less CLm split intein. a) PTS was only observed between P_N_ and P_C_ of the cognate split intein pairs, LCGC14 (**2P** and **8P**) and CLm (**10P** and **11P**), and not in any of the cross‐splicing combinations. b) Analysis of all possible pairwise combinations of the LCGC14 and CLm split intein precursors. Shown are Coomassie‐stained SDS‐PAGE gels of all possible PTS reactions performed at 37 °C, using each precursor at 10 µM. SP = splice product. Uncropped SDS‐PAGE images are shown in Figure S12.

We next asked whether the LCGC14 intein is orthogonal to and could be used in combination with the CLm split intein.^[^ ^19^ ^]^ Since the two efficiently splicing CLm^[^ ^19^ ^]^ and CL^[^ ^45^ ^]^ inteins were both engineered on the basis of the same cysteine‐less Aes123 PolB1 (Aes) intein^[^ ^45^ ^]^ we did not expect them to be orthogonal. We confirmed this assumption in cross‐splicing experiments (Figure S8). To our delight, when mixing the LCGC14 precursors **2P** and **8P** in both possible combinations with the CLm precursor constructs MBP‐CLm^N^‐H_6_ (**10P**) and Aes^C^‐GFP (**11P**), no splice product formation could be detected even after 12 h, indicating strict orthogonality (Figure 5). Notably, no cleavage products were observed, indicating precursor stability in the presence of the mismatched intein partner.

**Figure 6:**
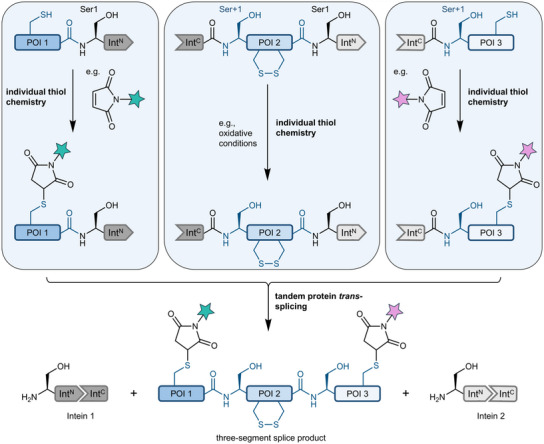
Scheme of three‐segment protein labeling. Three segments of a POI are assembled by tandem PTS using the orthogonal and cysteine‐less CLm and LCGC14 inteins. Each segment can be individually treated with regard to its thiol chemistry (for example, by thiol‐bioconjugation or by maintaining oxidative conditions).

To demonstrate the added value of two orthogonal, cysteine‐less split inteins, we developed a novel three‐segment tandem PTS scheme that allows selective chemical treatment of each protein segment prior to their assembly into a single polypeptide (Figure 6). For example, the individual segments could be selectively treated to introduce chemical labels by cysteine‐specific bioconjugation or to preserve native disulfide bonds.

Specifically, we were interested in the generation of a Förster resonance energy transfer (FRET) sensor with two thiol‐conjugated dyes of a large, trimodular non‐ribosomal peptide synthetase (NRPS). Such sensors enable the investigation of conformational changes resulting from altered interactions among the individual domains embedded within each of the NRPS modules.^[^ ^54^, ^55^, ^56^ ^]^ However, the preparation of such constructs is challenging due to the large protein sizes (each module is about 120 kDa) and the high number of endogenous cysteine residues, which would require extensive site‐directed mutagenesis for cysteine removal, associated with the risk of activity losses if expressed as a single protein.

**Figure 7:**
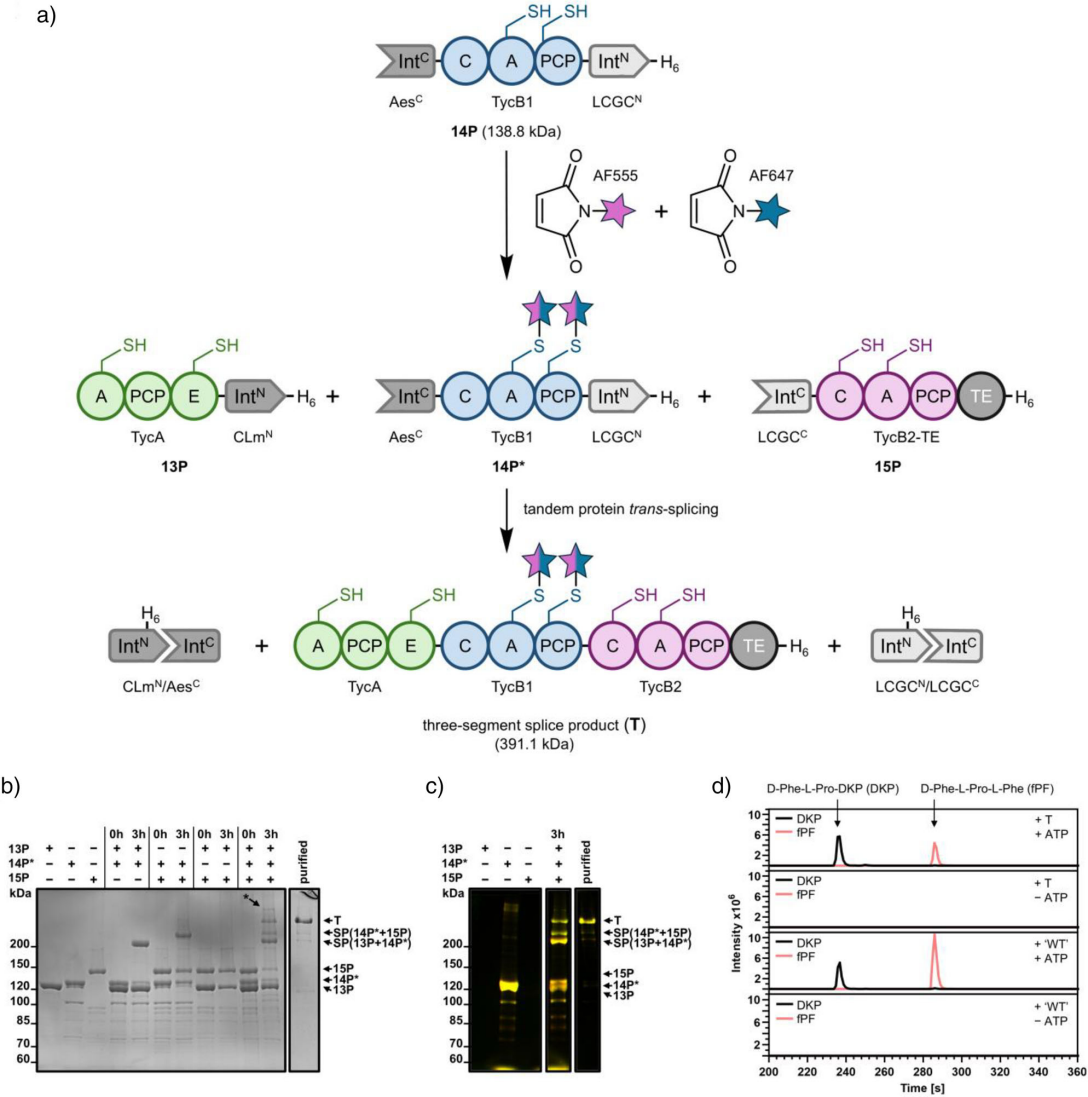
Three‐segment protein labeling to generate a fluorescently labeled trimodular NRPS. a) Reaction scheme to generate a trimodular NRPS specifically labeled in the central segment. Two accessible cysteines in the central segment (TycB1) were stochastically labeled with FRET donor and acceptor dyes and subsequently quenched with 2 mM DTT. Note that additional cysteines, in total 5 in the N‐terminal segment (TycA) and 10 in the C‐terminal segment (TycB2‐TE), remained unaffected by the thiol‐bioconjugation reaction. During the tandem PTS reaction, the 4′‐phosphopantetheinyltransferase Sfp and coenzyme A were added to convert the NRPS modules into their active holo‐states. b) Analysis of the PTS reactions using a Coomassie‐stained SDS‐PAGE gel. Precursor proteins were mixed at equimolar concentrations (4.5 µM) in the indicated combinations and incubated at 25 °C for 3 h. The three‐segment splice product (**T**) was purified by SEC. c) Fluorescence scan of selected lanes of the SDS‐PAGE analysis shown in b). Note that only the central (TycB2) segment is labeled. The yellow color results from the overlay of the AF555 and AF647 channels and indicates the stoichiometric ratio between both fluorophores. d) Product formation assay of the purified splice product holo‐TycA‐TycB1‐TycB2‐TE (**T**) and the imitated wildtype (“WT”) set‐up with separate proteins holo‐TycA (**16P**) and holo‐TycB1‐TycB2‐TE (**17P**), amino acid substrates, and ATP. Shown are extracted ion chromatograms. The cyclic diketopiperazine (DKP) dipeptide is a known side product resulting from premature cleavage of the NRPS‐bound thioester intermediate.^[^ ^58^ ^]^ Calculated MWs for the splice products of the single PTS reactions are SP(13P+14P*) = 256.9 kDa and SP(14P*+15P) = 269.7 kDa (without the masses of the fluorophores). (*) denotes an unidentified contamination. C = condensation domain, A = adenylation domain, PCP = peptidyl carrier protein domain, E = epimerization domain, and TE = thioesterase domain. Uncropped SDS‐PAGE images are shown in Figure S12.

As shown in Figure 7a, we selected TycB1, the second module of the tyrocidine NRPS, with two strategically placed cysteines for fluorophore bioconjugation.^[^ ^55^ ^]^ This central segment was prepared as a fusion protein with the Aes^C^ and LCGC^N^ intein fragments (construct **14P**). As splice partners, TycA and TycB2, as the first and third modules of the tyrocidine NRPS,^[^ ^57^ ^]^ were fused with the complementary CLm^N^ and LCGC^C^ fragments, respectively (**13P** and **15P**). These two modules retained their native cysteines (5 and 10, respectively), and TycB2 carried a fused thioesterase domain (TE) for peptide offloading from the NRPS template.^[^ ^58^ ^]^ Construct **14P** was stochastically labeled with Alexa Fluor (AF) 555 and AF647 maleimides to give 14P*. Following quenching of excess maleimides with 2 mM DTT, all three proteins, **13P**, 14P*, and **15P**, were mixed to initiate the tandem PTS reaction. In the same reaction, the 4′‐phosphopantetheine transferase (Sfp) and coenzyme A were added to convert the inactive apo‐modules into the active holo‐forms.^[^ ^59^ ^]^ Importantly, performing the thiol bioconjugation prior to mixing the proteins for the PTS reactions restricted the fluorophore labeling to the central TycB1 segment.

Figure 7b shows that the desired trimodular splice product TycA‐TycB1‐TycB2‐TE (**T**) was successfully formed in good yields, while control reactions with all possible combinations of only two intein precursors led to the formation of the expected dimodular splice products and confirmed the orthogonality between the CLm^N^ and LCGC^C^ fragments, respectively. Notably, the incomplete PTS observed for the reaction between 14P* and **15P** was not a result of a partially inactive LCGC^N^ precursor but must have resulted from the combination of the large TycB1 and TycB2‐TE exteins because control reactions with other cross‐combinations of LCGC precursor proteins showed virtually quantitative PTS for both partners (Figure S9). Subsequently, the fluorescently labeled holo‐TycA‐TycB1‐TycB2‐TE (**T**) was purified from the splice reaction via SEC with a total yield of 11% after splicing and purification (Figures 7b,c and S10). Incubation with ATP and the substrate amino acids L‐Phe (for TycA and TycB2) and L‐Pro (for TycB1) confirmed its enzymatic activity, as judged by the formation of the expected tripeptide D‐Phe‐L‐Pro‐L‐Phe (fPF) detected by LC‐MS (Figure 7d). Note that the observed D‐Phe‐L‐Pro‐diketopiperazine (DKP) is an expected by‐product resulting from uncatalyzed premature off‐loading of the D‐Phe‐L‐Pro‐thioester intermediate.^[^ ^58^ ^]^ In comparison to a non‐spliced control with proteins TycA (**16P**) and TycB1‐TycB2‐TE (**17P**), the tripeptide was obtained with a reduced yield of 38%, which is likely due to the short peptide linkers introduced as extein residues in the splicing reactions (1 and 14 aa, respectively).

As a second example for the new three‐segment protein labeling scheme, we decided to bioconjugate cysteine(s) in each of three protein segments with a different fluorophore. To this end, we exchanged the TycB1 sequence in protein **14P** with a 13 aa sequence (termed CysTag) including a single cysteine (construct **18P**) to insert a short, labeled peptide segment in the middle of a protein. Figure S11 shows that individual bioconjugation of **13P**, **18P**, and **15P** with AF555, fluoresceine, and AF647 dyes to give 13P*, 18P*, and 15P*, respectively, followed by maleimide quenching and tandem PTS resulted in the tripartite TycA‐CysTag‐TycB2‐TE with selective triple color labeling. Together, these examples clearly underline the potential of the new LCGC14 intein for new schemes in protein preparation and modification.

## Conclusion

To improve the identification of new efficiently splicing split inteins, we introduced a novel pre‐selection method solely based on amino acid sequences. None of the activity‐related important features of split inteins identified from genomic or metagenomic sequences could be recognized so far from their sequence. Our procedure builds on previous findings that a β‐sheet‐dependent aggregate formation of the Int^N^ precursor limits the splicing efficiency of many split inteins.^[^ ^19^ ^]^ We show that computational prediction of the aggregation propensity using a web‐based tool provides an alternative to elaborate biochemical characterization of each individual split intein. Our method correctly predicted the aggregation propensities of three new cysteine‐less split intein candidates, with the Int^N^ fragment of the LCGC14 split intein found to have low aggregation propensity. Biochemical analysis showed an exclusively monomeric nature of an LCGC^N^ precursor protein and its virtually complete splicing efficiency. In contrast, for the two other candidates, a high aggregation propensity was predicted and biochemically confirmed.

The newly identified and characterized LCGC14 intein represents the first efficiently splicing cysteine‐less split intein orthogonal to the CLm or CL split inteins,^[^ ^19^, ^45^ ^]^ which were both derived from the same aggregation‐prone Aes split intein.^[^ ^45^ ^]^ The three other cysteine‐less and biochemically characterized split inteins reported to date^[^ ^20^, ^60^, ^61^ ^]^ are limited by several drawbacks, including the requirement of a renaturation step,^[^ ^60^ ^]^ poor splicing activity,^[^ ^60^, ^61^ ^]^ activity restricted to elevated temperatures (>50 °C),^[^ ^61^ ^]^ and high propensity to undesired cleavage side reactions^[^ ^20^ ^]^ that preclude their utility as robust and broadly applicable tools.

We demonstrated the expanded scope in protein engineering and modification enabled by two such orthogonal, cysteine‐less split inteins. In a new tandem PTS scheme, each of three protein segments can be individually treated regarding the chemistry of their cysteine residues before being spliced together (Figure 6). We used one possible variation of this scheme to site‐selectively label only the cysteine pair in the central segment of a trimodular NRPS to introduce donor and acceptor fluorophores for a FRET sensor. This and other similarly designed new NRPS FRET sensors, each consisting of multiple catalytic and carrier domains within their modules, will be invaluable in future studies to monitor and disentangle the conformational changes associated with the complex and dynamically changing domain–domain interactions during the catalytic steps of nonribosomal peptide assembly.^[^ ^54^, ^55^, ^56^, ^62^ ^]^ Many other applications for the simultaneous use of two cysteine‐less split inteins are conceivable, for example, to modify proteins under oxidizing conditions, such as in the extracellular environment, and in the absence of reducing agents. Two orthogonal cysteine‐less split inteins would also be a requirement to adapt the recently reported protein editing technology^[^ ^63^, ^64^ ^]^ to oxidizing environments. More generally, efficiently splicing split inteins expand the toolbox of click biology,^[^ ^65^ ^]^ alongside other approaches to posttranslationally link proteins or peptides.^[^ ^12^, ^13^, ^14^ ^]^

While our new computational analysis approach was very successful in this study to correctly predict a highly efficient split intein, some potential caveats to the generality of this method should be noted. The misfolding of the Int^N^ fragment into soluble aggregated, amyloid‐like structures appears to stem from an intrinsic folding conflict mostly concentrated within the N2 lobe, which is created by the presence of both disorder‐ and order‐promoting residues.^[^ ^19^ ^]^ Since these sequence properties lay the foundation for the assembly mechanism of typically split Int^N^ and Int^C^ fragments, most such split inteins are likely to exhibit the tendency to aggregate formation under certain conditions. Additional parameters likely to influence the aggregation propensity are the extein sequence and the Int^N^ precursor concentration. For example, the *Npu* DnaE intein is known for its generally high splicing efficiency,^[^ ^23^ ^]^ consistent with a low N2/N1 score ratio in our analysis. However, it has also been reported to form inactive, soluble aggregates in the context of certain extein sequences.^[^ ^19^, ^23^ ^]^

Another point to consider is based on the observation that artificially split inteins, generated from continuous inteins, show a higher N2/N1 aggregation score ratio than naturally split inteins.^[^ ^19^ ^]^ The tolerance of contiguous inteins for such high N2 scores can be explained by the direct link between their Int^N^ and Int^C^ fragments (translated as a single polypeptide), which kinetically prevents the misfolding into aggregates observed for isolated Int^N^ precursors. However, naturally split inteins are believed to have originated from continuous inteins.^[^ ^66^ ^]^ We therefore assume that a gene‐splitting event in the past was likely followed by evolutionary pressure to accumulate mutations that help avoid aggregation of the Int^N^ fragment by decreasing the N2 aggregation score as well as possibly increasing the N1 aggregation score to counterbalance the overall folding propensity. This process would result in the lower aggregation behavior and lower N2/N1 aggregation scores seen for contemporary, naturally split inteins.^[^ ^19^ ^]^ If true, the evolutionary age of the splitting event should inversely correlate with the Int^N^ aggregation propensity. Additional organism‐specific factors influencing the evolution of the aggregation properties of split inteins could be intracellular expression levels of the Int^N^ and Int^C^ precursors as well as the degree to which their expression is spatially and temporally synchronized to avoid prolonged exposure of the Int^N^ precursor in the absence of its Int^C^ partner.

Together, despite these potential caveats, our new predictive metric based on the N2/N1 aggregation score represents the first sequence‐based pre‐selection method for the identification of new split inteins, addressing lower efficiency caused by aggregation as a key general limitation of split inteins. While additional data may further refine this approach, we propose that it holds significant potential to help expand the repertoire of efficiently splicing split inteins.

## Supporting Information

The authors have cited additional references within the Supporting Information.^[^ ^67^, ^68^ ^]^

## Conflict of Interests

The authors declare no conflict of interest.

## Supporting information



Supporting Information

## Data Availability

The plasmids pCH308 and pCH301 encoding the LCGC14 N‐ and C‐terminal precursors **2P** and **8P**, respectively, have been deposited at Addgene under the accession codes: 245527 and 245528.
