# Supplementary material for: Three‐Segment Protein Labeling Using a Highly Efficient and Cysteine‐Less Split Intein Identified with Computational Prediction of Aggregation Properties
Source: Angew Chem Int Ed Engl. 2025 Oct 23;64(51):e202515821. doi: 10.1002/anie.202515821 (PMC12707363; doi:10.1002/anie.202515821)
Supplement: Supplementary file 1 — Supporting Information [file ANIE-64-e202515821-s001.pdf]

Supplementary Information

for

Three-segment protein labeling using a new highly efficient and cysteine-less split intein identified with computational prediction of aggregation properties

Christoph Humberg,[1#] Jonas Kröger,[1#] Shmuel Pietrokovski,[2*] Henning D. Mootz[1*]

[1]Institute of Biochemistry, University of Münster, Corrensstraße 36, 48149 Münster, Germany

[2]Department of Molecular Genetics, Weizmann Institute of Science, Rehovot 76100, Israel

# these authors contributed equally to this work

*corresponding authors: Henning.Mootz@uni-muenster.de,

shmuel.pietrokovski@weizmann.ac.il

Table of Contents Page

Experimental Section

Identification of new split intein candidates from data bases **S2**

Recombinant gene expression and protein purification **S2**

Protein trans-splicing assay **S3**

Densitometric analysis and determination of protein trans-splicing rate constants **S3**

Analytical size exclusion chromatography **S4**

Mass spectrometry analysis **S4**

Tandem LC-MS analysis of tryptic peptides **S5**

Three-segment splicing with individual chemical labeling **S6**

NRPS peptide formation assay **S6**

Bioinformatic analyses **S7**

Supplementary Tables and Figures

**Table S1**	List of purified recombinant protein constructs and their expression plasmids.	**S8**
**Table S2**	List of the determined splice kinetics.	**S8**
**Table S3**	List of sequences of recombinantly produced proteins.	**S9**
**Table S4**	List of the split intein nucleotide sequences used in the respective expression plasmids	**S12**
**Figure S1**	Structural prediction of the three investigated cysteine-less split inteins.	**S13**
**Figure S2**	Sequence-based aggregate prediction of commonly used native and artificially split inteins.	**S14**
**Figure S3**	Computational prediction and experimental verification of Int^N precursor aggregation.	**S15**
**Figure S4**	LC-MS analysis of the PTS reaction using the LCGC14 precursors conforming correct product formation	**S16**
**Figure S5**	Analysis of the unknown double band in Figure 4b using the LCGC14 precursors by LC-MS/MS**.**	**S17**
**Figure S6**	Splice assays with Int^N precursor (P_N) in excess to analyze the Int^C precursor (P_C) activity.	**S17**
**Figure S7**	Splice activity of the native cysteine-independent split inteins without prior SEC purification	**S18**
**Figure S8**	Cross reactivity between the cysteine-less CLm and CL inteins.	**S19**
**Figure S9**	Analysis of PTS activity of LCGC intein precursors Aes^C-TycB1-$LCGC^N$ (**14P**) and $LCGC^C$-TycB2-TE (**15P**).	**S19**
**Figure S10**	Purification of the three-segment splice product **13P-14P*-15P** (**T**) as shown in Figure 7 by size-exclusion chromatography (SEC).	**S20**
**Figure S11**	Three-segment protein labeling to generate a selectively labeled triple-color protein.	**S21**
**Figure S12**	Unprocessed SDS-PAGE images of the figures shown in the main text	**S22**
**Figure S13**	Unprocessed SDS-PAGE images of the figures shown in the supplementary information	**S23**
**References**		**S24**

Experimental Section

Identification of new split intein candidates from data bases

The cysteine-less split intein proteins were identified as previously described[1-2] by sequence searches of public nucleotide and protein sequence databases. NCBI database accessions and coordinates of the split inteins with their protein hosts are: CP21 PolB1: NC_019507.1:179333-177122 and NC_019507.1:176131-175225, LCGC14 PolB2: LAZR01010553.1:3840-2573 and LAZR01010553.1:1-873, and GOM-OPS-1 PolB2: JQIA01000298.1:3216-5393 and JQIA01000298.1:5441-6382.

Recombinant gene expression and protein purification

Plasmid-encoded constructs were generally expressed in *E. coli* LOBSTR BL21 (DE3) Gold, or in *E. coli* EC100 (ΔentD)[3] for **14P**, grown at 37 °C, and protein expression was induced at 18 °C overnight by addition of either IPTG (0.4 mM) or L-arabinose (0.2% (w/v)), depending on the plasmid (split intein nucleotide sequences are listed in Table S4). Cells were ruptured using an Emulsiflex C5 (Avestin) or by sonication (10 s on/ 15 s off) for 12 min. Proteins fused to a hexa-histidine tag were purified by Ni-NTA affinity chromatography at 4 °C using gravity flow columns (Cube Biotech) in Ni-NTA buffer (50 mM Tris, 300 mM NaCl, 20 mM imidazole, pH 8.0) and eluted with the same buffer containing 250 mM imidazole. Afterwards, proteins were dialyzed against SP buffer (50 mM Tris, 300 mM NaCl, 1 mM EDTA, pH 7.0) overnight at 4 °C.

Protein **11P** was produced bearing an N-terminal H_6-Smt3 tag as previously described.[4] The cells were lysed by sonication and purified by Ni-NTA chromatography as described above. After further purification the eluted protein was treated with 500 nM His_6-tagged Ulp1 for 30 min at 8 °C. After passing the protein mixture over Ni-NTA resin to remove Ulp1 and the cleaved Smt3 tag the desired product was obtained in the flowthrough.

Proteins **13P**, **14P** and **15P** were produced and cells were lysed by sonication as stated above. The cell lysate was filtered using a syringe filter (0.2 µm) and mixed with imidazole (c_F =

30 mM). The protein was then purified via affinity chromatography using the ÄKTA pure 25 system with the HisTrap HP 1 mL column, a flow rate of 1 mL/min and a gradient elution, starting at an imidazole concentration of 30 mM for 10 CV, increasing to 60 mM imidazole for 4 CV, increasing to 300 mM imidazole for 1 CV and staying at 300 mM for 10 CV. Buffers used were NRPS assay buffer (50 mM HEPES, 100 mM NaCl, 10 mM $MgCl_2$, 1 mM EDTA) and NRPS elution buffer (NRPS assay buffer + 300 mM imidazole). The eluted protein was purified via size exclusion chromatography using the ÄKTA pure 25 system with a Superdex® 200 Increase 10/300 GL column and a flow rate of 0.75 mL/min with NRPS assay buffer.

Protein concentrations were determined using the calculated extinction coefficient at 280 nm. The identity of the products was confirmed by ESI-MS and the purity was assessed by SDS-PAGE or analytical RP-HPLC.

Protein *trans*-splicing assay

Reactions were started by mixing the N- and C-terminal intein precursor proteins in SP buffer (50 mM Tris, 300 mM NaCl, 1 mM EDTA, pH 7.0) at indicated concentrations at 37 °C and in absence of reducing agents. At indicated time points aliquots were removed and the reaction was stopped by adding 4x SDS-PAGE loading buffer (500 mM Tris/HCl, 8% (w/v) SDS, 40% (v/v) glycerine, 20% (v/v) β-mercaptoethanol, 5 mg/L bromophenol blue, pH 6.8) and boiling (95 °C, 5 min). Splice product formation was analyzed by SDS-PAGE.

Densitometric analysis and determination of protein *trans*-splicing rate constants

Coomassie-stained bands were analyzed using Gel Analyzer (v2010a) and normalized to the corresponding molecular weight. Normalized intensities were used to calculate the ratio *x* of splice product to the precursor protein used in limiting amount to determine the splice yield as follows.

$$P(\%) = \frac{(100 \times x)}{100 + x} \qquad (1)$$

To determine the splice rate the splice product formation was treated as a pseudo-first-order reaction with one of the precursors given in three-fold molar excess and plotted against time. The following single exponential function was fitted to the data using GraphPad Prism (v8).

$$[P]_t = P_{max}(1 - e^{-k_{total}t}) \quad (2)$$

where P is the normalized intensity of the splice product and k_{total} describes the pseudo-first-order rate equation of the protein trans-splice reaction. The variable t is the reaction time in seconds and P_{max} is a normalization factor which represents the fraction of active precursor protein.

Analytical size exclusion chromatography

Analytical gel filtration was done using a 1260 infinity LC system (Agilent) equipped with an AdvanceBio SEC 200Å 1.9 µm, 4.6 × 300 mm (Agilent) column at flowrates of 0.35 mL/min. The samples (**1P**: 60 µM, **2P**: 130 µM, **3P**: 75 µM, **4P**: 115 µM, **5P**: 240 µM, **6P**: 60 µM) were adjusted to a concentration of 10 µM and incubated for 24 h at 15 °C prior to SEC analysis to exclude concentration-dependent artefacts in the aggregate proportion. Analysis was then carried out in SP buffer at 15 °C by injecting 5 µL of the sample. The obtained UV profiles at 280 nm were normalized to the highest peak to determine the aggregate/monomer ratio by the integrated peak area using GraphPad Prism (v8).

The hydrodynamic radius and the corresponding molecular weight (MW) of globular proteins was estimated using a linear calibration plot of retention volume vs. log MW, obtained with the standard globular molecular weight markers β-amylase (MW = 200 kDa), alcohol dehydrogenase (MW = 150 kDa), bovine serum albumin (MW = 66 kDa), carbonic anhydrase (MW = 29 kDa) and cytochrome c (MW = 12.4 kDa) according to the method described elsewhere.

Mass spectrometry analysis

Mass analysis of intact proteins was performed using an UltiMate™ 3000 RS system (Thermo Fisher Scientific GmbH) connected to a maXis II UHR-qTOF mass spectrometer (Bruker

Daltonik GmbH) with a standard ESI source (Apollo, Bruker Daltonik GmbH). Samples were acidified using a 10% formic acid solution to reach a pH 2-3 and centrifuged (20,000 x g, 3 min). According to the protein concentration, an appropriate volume of the supernatant was loaded on a C4 column (Advance Bio RP-mAb C4, 2.1 mm x 50 mm, 3.5 µm, Agilent Technologies) at a flow rate of 0.6 mL/min in 5% eluent B (eluent A: 0.1% formic acid in water; eluent B: 0.1% formic acid in acetonitrile). After a desalting period of 7 minutes at 5% B, a steep gradient was applied (5-60% B in 2 min). MS settings: capillary voltage 4500 V, endplate offset 500 V, nebulizer 5.0 bar, dry gas 9.0 L/min, dry T = 200 °C, mass range m/z 300-3000. Data were analyzed with DataAnalysis (v5.3) (Bruker Daltonik GmbH) and deconvolution was performed using the MaxEnt algorithm implemented in the software.

Tandem LC-MS analysis of tryptic peptides

For tryptic protein in-gel digestions, the protein mixture was mixed with 4x SDS loading dye (250 mM Tris/HCl, pH 6.8, 8% (w/v) SDS, 40% (v/v) glycerine, 20% (v/v) β-mercaptoethanol, 0.2% (w/v) bromophenol blue) and heated to 95 °C for 5 min. Following separation by SDS-polyacrylamide gel electrophoresis and Coomassie brilliant blue staining, protein bands were excised, destained by 50% (v/v) EtOH in H_2O, 0.1% (v/v) TFA at 60 °C overnight, washed and dried with acetonitrile and by vacuum concentration. Trypsin digestion was performed at 37 °C using 400 ng pre-warmed trypsin (Promega) in 50 mM ammonium carbonate supplemented with 0.01% ProteaseMax (Promega) for 6 h. Afterwards, the supernatant (20 µL) was collected and acidified with 0.1% formic acid as final concentration and analyzed by tandem LC-MS without further dilution.

Tandem MS analysis of tryptic peptides (LC-MS2) was performed using an UltiMate™ 3000 RS LC nano system (Thermo Fisher Scientific GmbH) connected to a maXis II UHR-qTOF mass spectrometer with a nano-ESI source (CaptiveSpray with nanoBooster, Bruker Daltonik GmbH). 3.5 µL of each sample were loaded on a C18 trapping column (Acclaim PepMap™ 100, 5 µm, 100 Å, ID 100 µm x L 20 mm, Thermo Fisher Scientific GmbH) at a flow rate of 20 µL/min in 2% eluent B (eluent A: 0.1% formic acid in water; eluent B: 0.1% formic acid in

acetonitrile). After 10 min of washing at 2% B, a 45-minute gradient (5 to 50% B, flow rate 500 nL/min) was applied for the separation on a C18 nano column (PepSep TWENTY-FIVE C18, ID 150 µm x L 250 mm, 1,5 µm, Bruker Daltonik GmbH). MS settings: capillary voltage 1600 V, mass range: m/z 150-2200. MS survey scans were performed with a cycle time of 2.5 s. After each survey scan, the 10-20 most abundant precursor ions with z > 1 were selected for fragmentation using collision-induced dissociation. MS/MS summation time was adjusted depending on the precursor intensity, the precursor isolation window and the collision energy were depending on the precursor m/z and charge. DataAnalysis (v5.3) (Bruker Daltonik GmbH) was used for chromatogram processing and fragment spectra isolation. The resulting mgf files were analyzed using ProteinScape (v4.2) (Bruker Daltonik GmbH) as a front-end for searches against the SWISS-PROT databases on a Mascot server (Mascot 2.5, Matrix Science Ltd.)

Three-segment splicing with individual chemical labeling

Thiol bioconjugation of protein **14P** was performed by first reducing the purified protein (9.3 µM) using TCEP (10 eq.) for 30 min at 18 °C before adding the AlexaFluor555 C_2 maleimide (5 eq.) and AlexaFluor 647 C_2 maleimide (10 eq.) for another 35 min at 18 °C. The labeling reaction was quenched by the addition of DTT (2 mM) for 5 min at 18 °C. Subsequently, the two PTS reactions were triggered by adding proteins **13P** and **15P** to give all three split intein precursor in equimolar concentrations (4.5 µM each). Simultaneously, 4'-phosphopantheteinlyation was carried out by purified Sfp (0.05 eq.), CoA (50 eq.), $MgCl_2$ (10 mM), and TCEP (1 mM). The reaction mixture was incubated for 3 h at 25 °C.

The splice product was purified via size exclusion chromatography using the ÄKTA purifier 10 system and the columns Superdex 200 Increase 10/300 GL and HiLoad 16/600 Superdex 200 prep grade connected in series with a flow rate of 0.4 mL/min with NRPS assay buffer.

NRPS peptide formation assay

The purified splice product holo-TycA-TycB1-TycB2-TE (**T**; 5 µM) was incubated with L-Phe (2 mM), L-Pro (1 mM), ATP (5 mM) and $MgCl_2$ (20 mM) for 1 h at 25 °C, followed by thermal

inactivation at 85 °C for 30 min. The denatured protein was separated from the peptide containing solution by centrifugation (3 min, 20,000 × *g*). The supernatant was mixed with formic acid to a final concentration of 1% (v/v) before centrifugation (3 min, 20,000 × *g*) and then used for MS analysis. As control, the imitated wildtype set-up consisting of the separate proteins holo-TycA (**16P**) and holo-TycB1-TycB2-TE (**17P**) was used.

MS analysis of the resulting peptides was performed with the same setup as described for intact proteins, with the following changes: The column was changed to the ZORBAX SB-C18 RR HT column (80 Å, 1.8 µm, L 50 mm x D 3 mm, Agilent Technologies, Waldbronn, Germany). The gradient was changed to 1% to 40% B in 6 min, followed by 40% to 95% in 1 min. Nebulizer pressure was changed to 3.0 bar, the dry gas flow to 8.0 L/min at T=250 °C.

Bioinformatic analyses

Aggregate-forming regions were predicted by the sequence-based web server AMYLPRED2 which employs a consensus of different methods to predict amyloid fibril formation.[5] Note that the method AmyloidMutants method of the server was not used due to connection errors to it by the server.

Structural data or, where unavailable, structural predictions were used to define the N1 and N2 lobes of the Int^N fragment, with the boundary set at the beginning of β-strand 7. Aggregation propensity scores (ranging from 2 – 10 per residue) were obtained from the AMYLPRED2 server,[5] and for each lobe the average score was calculated as the sum of scores (Σ) divided by n, where n is the number of residues in the respective lobe. The predictive aggregation metric was then defined as the ratio of the N2 lobe average score to the N1 lobe average score.

Protein structure prediction was done using AlphaFold3.[6] The multiple sequence alignment was done using multiple sequence comparison by log-expectation.[7]

Supplementary Tables

Table S1 List of purified recombinant protein constructs and their expression plasmids.

Protein	Construct	MW* [kDa]	Encoding Plasmid	Vector System	Reference
**1P**	MBP-CP21^N(C43S)-H_6	57.7	pCH160	pMAL-c2x	this work
**2P**	MBP-LCGC^N(C24S)-H_6	58.2	pCH308	pMAL-c2x	this work
**3P**	MBP-GOM^N(C57S)-H_6	57.8	pCH309	pMAL-c2x	this work
**4P**	MBP-CP21^N-H_6	57.7	pTT77	pMAL-c2x	this work
**5P**	MBP-LCGC^N-H_6	58.2	pCH300	pMAL-c2x	this work
**6P**	MBP-GOM^N-H_6	57.8	pCH304	pMAL-c2x	this work
**7P**	CP21^C-diSUMO-H_6	27.3	pCH162	pST Duett 51F	this work
**8P**	LCGC^C-sfGFP-H_6	32.4	pCH301	pBAD	this work
**9P**	GOM^C-sfGFP-H_6	32.9	pCH305	pBAD	this work
**10P**	MBP-CLm^N-H_6	58.2	pCH145	pMAL-c2x	Ref[4]
**11P**	Aes^C-sfGFP (**Precursor**: H_6-smt3-Aes^C-sfGFP)	31.3	pCH196	pET28b	Ref[4]
**12P**	SBP-CL^C-Trx-H_6	33.8	pTT43	pET16b	Ref[8]
**13P**	TycA-D-CLm$^{N(}$V84A)-H_6	137.5	pJK60	pET28a	this work
**14P**	Aes^C-TycB1(C98A,C154S,C430A, C440A,A553C,C725S,C768A,C907A, C1021A)-SGVCTEDTD-LCGC^N(C24S)-H_6	138.8	pJK84	pTrc99a	this work
**15P**	LCGC^C-SAYGG-TycB2-TE-H_6	151.0	pJK83	pTrc99a	this work
**16P**	SBP-TycA	126.9	pJR89	pET28a	Ref[9]
**17P**	TycB1-TycB2-TE-H_6	264.0	pJK19	pTrc99a	this work
**18P**	H_6-Smt3-Aes^C-SVYGTSCSTGDTD-LCGC^N-SBP	38.0	pJK91	pET28a	this work

*MW = molecular weight.

Table S2 List of the determined splice kinetics.

Split intein	native constructs	cysteine-less constructs
CP21 PolB1	6.4 ± 0.5 x 10^{-3} s^{-1}	19.4 ± 5.1 x 10^{-3} s^{-1}
LCGC14 PolB2	1.53 ± 0.24 x 10^{-3} s^{-1}	1.32 ± 0.09 x 10^{-3} s^{-1}
+1 mM TCEP	2.04 ± 0.22 x 10^{-3} s^{-1}	
GOM-OPS-1 PolB2	0.35 ± 0.04 x 10^{-3} s^{-1}	0.35 ± 0.03 x 10^{-3} s^{-1}

Table S3 List of sequences of recombinantly produced proteins.

Protein	Construct	Sequence
**1P**	MBP-Linker-DTD-<br>CP21^{N}(C43S)-H$_6$	MKTEEGKLVIWINGDKGYNGLAEVGKKFEKDTGIKVTVEHPDKLEEKFPQVAATGDGPDIIFW<br>AHDRFGGYAQSGLLAEITPDKAFQDKLYPFTWDAVRYNGKLIAYPIAVEALSLIYNKDLLPNPP<br>KTWEEIPALDKELKAKGKSALMFNLQEPYFTWPLIAADGGYAFKYENGKYDIKDVGVDNAGAK<br>AGLTFLVDLIKNKHMNADTDYSIAEAAFNKGETAMTINGPWAWSNIDTSKVNYGVTVLPTFKG<br>QPSKPFVGVLSAGINAASPNKELAKEFLENYLLTDEGLEAVNKDKPLGAVALKSYEEELAKDP<br>RIAATMENAQKGEIMPNIPQMSAFWYAVRTAVINAASGRQTVDEALKDAQTNSSSNNNNNNN<br>NNNLGIEGRISEFYNDTDSVVGDSIIKVNGKNIKIEDFYDSIKVDPIVTKSGNNVKLVDNSFTESV<br>NKNLQIETKKINYIMKHKVKKEFFKIKVNNKEVVVTEDHSIMVLRNSELIEVKPRDIKNGDLIILND<br>GSHHHHHH
**2P**	MBP-Linker-DTD-<br>LCGC^{N}(C24S)-H$_6$	MKTEEGKLVIWINGDKGYNGLAEVGKKFEKDTGIKVTVEHPDKLEEKFPQVAATGDGPDIIFW<br>AHDRFGGYAQSGLLAEITPDKAFQDKLYPFTWDAVRYNGKLIAYPIAVEALSLIYNKDLLPNPP<br>KTWEEIPALDKELKAKGKSALMFNLQEPYFTWPLIAADGGYAFKYENGKYDIKDVGVDNAGAK<br>AGLTFLVDLIKNKHMNADTDYSIAEAAFNKGETAMTINGPWAWSNIDTSKVNYGVTVLPTFKG<br>QPSKPFVGVLSAGINAASPNKELAKEFLENYLLTDEGLEAVNKDKPLGAVALKSYEEELAKDP<br>RIAATMENAQKGEIMPNIPQMSAFWYAVRTAVINAASGRQTVDEALKDAQTNSSSNNNNNNN<br>NNNLGIEGRISEFDTDSVDADTIIKTNYGEMTIENLFKSSSIKGPSWAIDDQEFTIYDQIQILTYDP<br>KTNEEIYRPFEYVYRHKVSKPRWKIIDENGNEIILTNDHSVMIERDGKLIEAKPSEINPDTDILITI<br>GEGSHHHHHH
**3P**	MBP-Linker-DTD-<br>GOM^{N}(C57S)-H$_6$	MKTEEGKLVIWINGDKGYNGLAEVGKKFEKDTGIKVTVEHPDKLEEKFPQVAATGDGPDIIFW<br>AHDRFGGYAQSGLLAEITPDKAFQDKLYPFTWDAVRYNGKLIAYPIAVEALSLIYNKDLLPNPP<br>KTWEEIPALDKELKAKGKSALMFNLQEPYFTWPLIAADGGYAFKYENGKYDIKDVGVDNAGAK<br>AGLTFLVDLIKNKHMNADTDYSIAEAAFNKGETAMTINGPWAWSNIDTSKVNYGVTVLPTFKG<br>QPSKPFVGVLSAGINAASPNKELAKEFLENYLLTDEGLEAVNKDKPLGAVALKSYEEELAKDP<br>RIAATMENAQKGEIMPNIPQMSAFWYAVRTAVINAASGRQTVDEALKDAQTNSSSNNNNNNN<br>NNNLGIEGRISEFDTDSVAFNSIIEIDGIKDTIESWFNKLAEEHGRHVDGEKEFTKISSLDLHTPT<br>YDVAYDSMVDKPLMTIYRHKIEKKMYTVTSVDGHSVTTTADHSLMVMRGGNIIEIIPTDILSGD<br>QLVIFEGSHHHHHH
**4P**	MBP-Linker-DTD-<br>CP21^{N}-H$_6$	MKTEEGKLVIWINGDKGYNGLAEVGKKFEKDTGIKVTVEHPDKLEEKFPQVAATGDGPDIIFW<br>AHDRFGGYAQSGLLAEITPDKAFQDKLYPFTWDAVRYNGKLIAYPIAVEALSLIYNKDLLPNPP<br>KTWEEIPALDKELKAKGKSALMFNLQEPYFTWPLIAADGGYAFKYENGKYDIKDVGVDNAGAK<br>AGLTFLVDLIKNKHMNADTDYSIAEAAFNKGETAMTINGPWAWSNIDTSKVNYGVTVLPTFKG<br>QPSKPFVGVLSAGINAASPNKELAKEFLENYLLTDEGLEAVNKDKPLGAVALKSYEEELAKDP<br>RIAATMENAQKGEIMPNIPQMSAFWYAVRTAVINAASGRQTVDEALKDAQTNSSSNNNNNNN<br>NNNLGIEGRISEFYNDTDSVVGDSIIKVNGKNIKIEDFYDSIKVDPIVTKSGNNVKLVDNCFTESV<br>NKNLQIETKKINYIMKHKVKKEFFKIKVNNKEVVVTEDHSIMVLRNSELIEVKPRDIKNGDLIILND<br>GSHHHHHH
**5P**	MBP-Linker-DTD-<br>LCGC^{N}-H$_6$	MKTEEGKLVIWINGDKGYNGLAEVGKKFEKDTGIKVTVEHPDKLEEKFPQVAATGDGPDIIFW<br>AHDRFGGYAQSGLLAEITPDKAFQDKLYPFTWDAVRYNGKLIAYPIAVEALSLIYNKDLLPNPP<br>KTWEEIPALDKELKAKGKSALMFNLQEPYFTWPLIAADGGYAFKYENGKYDIKDVGVDNAGAK<br>AGLTFLVDLIKNKHMNADTDYSIAEAAFNKGETAMTINGPWAWSNIDTSKVNYGVTVLPTFKG<br>QPSKPFVGVLSAGINAASPNKELAKEFLENYLLTDEGLEAVNKDKPLGAVALKSYEEELAKDP<br>RIAATMENAQKGEIMPNIPQMSAFWYAVRTAVINAASGRQTVDEALKDAQTNSSSNNNNNNN<br>NNNLGIEGRISEFDTDSVDADTIIKTNYGEMTIENLFKSCSIKGPSWAIDDQEFTIYDQIQILTYD<br>PKTNEEIYRPFEYVYRHKVSKPRWKIIDENGNEIILTNDHSVMIERDGKLIEAKPSEINPDTDILIT<br>IGEGSHHHHHH
**6P**	MBP-Linker-DTD-<br>GOM^{N}-H$_6$	MKTEEGKLVIWINGDKGYNGLAEVGKKFEKDTGIKVTVEHPDKLEEKFPQVAATGDGPDIIFW<br>AHDRFGGYAQSGLLAEITPDKAFQDKLYPFTWDAVRYNGKLIAYPIAVEALSLIYNKDLLPNPP<br>KTWEEIPALDKELKAKGKSALMFNLQEPYFTWPLIAADGGYAFKYENGKYDIKDVGVDNAGAK<br>AGLTFLVDLIKNKHMNADTDYSIAEAAFNKGETAMTINGPWAWSNIDTSKVNYGVTVLPTFKG<br>QPSKPFVGVLSAGINAASPNKELAKEFLENYLLTDEGLEAVNKDKPLGAVALKSYEEELAKDP<br>RIAATMENAQKGEIMPNIPQMSAFWYAVRTAVINAASGRQTVDEALKDAQTNSSSNNNNNNN<br>NNNLGIEGRISEFDTDSVAFNSIIEIDGIKDTIESWFNKLAEEHGRHVDGEKEFTKISSLDLHTPT<br>YDVAYDCMVDKPLMTIYRHKIEKKMYTVTSVDGHSVTTTADHSLMVMRGGNIIEIIPTDILSGD<br>QLVIFEGSHHHHHH
**7P**	CP21^{C}-SCY-Linker-<br>diSUMO-H$_6$	MIVTENFQVESLGIQELDVYDIEVDSNHNFFANDILVHNSCYVQGTENLYFQGADEKPKEGVK<br>TENNDHINLKVAGQDGSVVQFKIKRHTPLSKLMKAYAERQGLSMRQIRFRFDGQPINETDTPA<br>QLEMEDEDTIDVFQQQTGGKTENNDHINLKVAGQDGSVVQFKIKRHTPLSKLMKAYAERQGL<br>SMRQIRFRFDGQPINETDTPAQLEMEDEDTIDVFQQQTGGTHGGSHHHHHH
**8P**	LCGC^{C}-SAY-sfGFP-H$_6$	MVEKLKIQKIEKLEDFDNEYVYDISVDKETPYFFGNNILVHNSAYGTSKGEELFTGVVPILVELD<br>GDVNGHKFSVRGEGEGDATNGKLTLKFICTTGKLPVPWPTLVTTLTYGVQCFSRYPDHMKRH<br>DFFKSAMPEGYVQERTISFKDDGTYKTRAEVKFEGDTLVNRIELKGIDFKEDGNILGHKLEYNF<br>NSHNVYITADKQKNGIKANFKIRHNVEDGSVQLADHYQQNTPIGDGPVLLPDNHYLSTQSVLS<br>KDPNEKRDHMVLLEFVTAAGITHGGSHHHHHH
**9P**	GOM^{C}-SAY-sfGFP-H$_6$	MHERKYKLVDIAKVEEVKYTDEYVYDVVMFEPESPYFVANDILVHNSAYGTSKGEELFTGVVP<br>ILVELDGDVNGHKFSVRGEGEGDATNGKLTLKFICTTGKLPVPWPTLVTTLTYGVQCFSRYPD<br>HMKRHDFFKSAMPEGYVQERTISFKDDGTYKTRAEVKFEGDTLVNRIELKGIDFKEDGNILGH<br>KLEYNFNSHNVYITADKQKNGIKANFKIRHNVEDGSVQLADHYQQNTPIGDGPVLLPDNHYLS<br>TQSVLSKDPNEKRDHMVLLEFVTAAGITHGGSHHHHHH
**10P**	MBP-Linker-DTD-<br>CLm^{N}-H$_6$	MKTEEGKLVIWINGDKGYNGLAEVGKKFEKDTGIKVTVEHPDKLEEKFPQVAATGDGPDIIFW<br>AHDRFGGYAQSGLLAEITPDKAFQDKLYPFTWDAVRYNGKLIAYPIAVEALSLIYNKDLLPNPP<br>KTWEEIPALDKELKAKGKSALMFNLQEPYFTWPLIAADGGYAFKYENGKYDIKDVGVDNAGAK<br>AGLTFLVDLIKNKHMNADTDYSIAEAAFNKGETAMTINGPWAWSNIDTSKVNYGVTVLPTFKG<br>QPSKPFVGVLSAGINAASPNKELAKEFLENYLLTDEGLEAVNKDKPLGAVALKSYEEELAKDP<br>RIAATMENAQKGEIMPNIPQMSAFWYAVRTAVINAASGRQTVDEALKDAQTNSSSNNNNNNN<br>NNNLGIEGRISEFYIDTDSVVGDTIIDVSGKKMTIAEFYDSTPDVFMRRNDEARDWVKRVGGK<br>TSLSVNTYSGEVERKNINYIMKHKVKKRMHKIKAGGKEVIVTADHSVMVKRDGKIIDVKPTEMK<br>QTDRVVKWNLTGSHHHHHH

Table S3 List of sequences of recombinantly produced proteins (continued).

Protein	Construct	Sequence
**11P**	H_6-smt3-Aes^C-SVY-sfGFP	MGSSHHHHHHSSGLVPRGSHMASMSDSEVNQEAKPEVKPEVKPETHINLKVSDGSSEIFFKI<br>KKTTPLRRLMEAFAKRQGKEMDSLRFLYDGIRIQADQTPEDLDMEDNDIIEAHREQIGGSMIEF<br>IEFEIEDLGVMEIDVYDIEVDGNHNFFGNDILVHNSVYLNGTSKGEELFTGVVPILVELDGDVNG<br>HKFSVRGEGEGDATNGKLTLKFICTTGKLPVPWPTLVTTLTYGVQCFSRYPDHMKRHDFFKS<br>AMPEGYVQERTISFKDDGTYKTRAEVKFEGDTLVNRIELKGIDFKEDGNILGHKLEYNFNSHN<br>VYITADKQKNGIKANFKIRHNVEDGSVQLADHYQQNTPIGDGPVLLPDNHYLSTQSVLSKDPN<br>EKRDHMVLLEFVTAAGITHG
**12P**	SBP-CL^C-SVY-Trx-H_6	MDEKTTGWRGGHVVEGLAGELEQLRARLEHHPQGQREPGASGGGGSSSEARDWVKRVGG<br>KTSLSVNTYSGEVERKNINYIMKHTVKKRMFKIKAGGKEVIVTADHSVMVKRDGKIIDVKPTEM<br>KQTDRVVKWMLTGSHMIEFIEFEIEDLGVMEIDVYDIEVDGNHNFFGNDILVHNSVYLNGTGSD<br>KIIHLTDDSFDTDVLKADGAILVDFWAHWCGPCKMIAPILDEIADEYQGKLTVAKLNIDHNPGTA<br>PKYGIRGIPTLLLFKNGEVAATKVGALSKGQLKEFLDANLAGSEFRSHHHHHH
**13P**	TycA-D-CLm^N(V84A)-H_6	MVANQANLIDNKRELEQHALVPYAQGKSIHQLFEEQAEAFPDRVAIVFENRRLSYQELNRKAN<br>QLARALLEKGVQTDSIVGVMMEKSIENVIAILAVLKAGGAYVPIDIEYPRDRIQYILQDSQTKIVL<br>TQKSVSQLVHDVGYSGEVVVLDEEQLDARETANLHQPSKPTDLAYVIYTSGTTGKPKGTMLE<br>HKGIANLQSFFQNSFGVTEQDRIGLFASMSFDASVWEMFMALLSGASLYILSKQTIHDFAAFE<br>HYLSENELTIITLPPTYLTHLTPERITSLRIMITAGSASSAPLVNKWKDKLRYINAYGPTETSICAT<br>IWEAPSNQLSVQSVPIGKPIQNTHIYIVNEDLQLLPTGSEGELCIGGVGLARGYWNRPDLTAEK<br>FVDNPFVPGEKMYRTGDLAKWLTDGTIEFLGRIDHQVKIRGHRIELGEIESVLLAHEHITEAVVI<br>AREDQHAGQYLCAYYISQQEATPAQLRDYAAQKLPAYMLPSYFVKLDKMPLTPNDKIDRKAL<br>PEPDLTANQSQAAYHPPRTETESILVSIWQNVLGIEKIGIRDNFYSLGGDSIQAIQVVARLHSYQ<br>LKLETKDLLNYPTIEQVALFVKSTTRKSDQGIIAGNVPLTPIQKWFFGKNFTNTGHWNQSSVLY<br>RPEGFDPKVIQSVMDKIIEHHDALRMVYQHENGNVVQHNRGLGGQLYDFFSYNLTAQPDVQ<br>QAIEAETQRLHSSMNLQEGPLVKVALFQTLHGDHLFLAIHHLVVDGISWRILFEDLATGYAQAL<br>AGQAISLPEKTDSFQSWSQWLQEYANEADLLSEIPYWESLESQAKNVSLPKDYEVTDCKQKS<br>VRNMRIRLHPEETEQLLKHANQAYQTEINDLLLAALGLAFAEWSKLAQIVIHLEGHGREDIIEQA<br>NVARTVGWFTSQYPVLLDLKQTAPLSDYIKLTKENMRKIPRKGIGYDILKHVTLPENRGSLSFR<br>VQPEVTFNYLGQFDADMRTELFTRSPYSGGNTLGADGKNNLSPESEVYTALNITGLIEGGELV<br>LTFSYSSEQYREESIQQLSQSYQKHLLAIIAHCTEKKEVERTPSDFSVKGLQMEEMDDIFELLA<br>NTLRDSVVGDTIIDVSGKKMTIAEFYDSTPDVFMRRNDEARDWVKRVGGKTSLSVNTYSGEV<br>ERKNINYIMKHKVKKRMHKIKAGGKEAIVTADHSVMVKRDGKIIDVKPTEMKQTDRVVKWNLT<br>SSRSHHHHHH
**14P**	Aes^C-TycB1(C98A,C154S,C430A, C440A,A553C,C725S, C768A,C907A, C1021A)-SGVCTEDTD-LCGC^N(C24S)-H_6	MIEFIEFEIEDLGVMEIDVYDIEVDGNHNFFGNDILVHNSVFSKEQVQDMYALTPMQEGMLFHA<br>LLDQEHNSHLVQMSISLQGDLDVGLFTDSLHVLVERYDVFRTLFLYEKLKQPLQVVLKQRPIPI<br>EFYDLSAADESEKQLRYTQYKRADQERTFHLAKDPLMRVALFQMSQHDYQVIWSFHHILMDG<br>WSFSIIFDDLLAIYLSLQNKTALSLEPVQPYSRFINWLEKQNKQAALNYWSDYLEAYEQKTTLP<br>KKEAAFAKAFQPTQYRFSLNRTLTKQLGTIASQNQVTLSTVIQTIWGVLLQKYNAAHDVLFGSV<br>VSGRPTDIVGIDKMVGLFINTIPFRVQAKAGQTFSELLQAVHKRTLQSQPYEHVPLYDIQTQSV<br>LKQELIDHLLVIENYPLVEALQKKALNQQIGFTITAVEMFEPTNYDLTVMVMPKEELAFRFDYNA<br>ALFDEQVVQKLAGHLQQIADAVANNSGVELAQIPLLTEAETSQLLAKRTETAADYPAATMHEL<br>FSRQAEKTPEQVAVVFADQHLTYRELDEKSNQLARFLRKKGIGTGSLVGTLLDRSLDMIVGIL<br>GVLKAGGAFVPIDPELPCERIAYMLTHSRVPLVVTQNHLRAKVTTPTETIDINTAVIGEESRAPI<br>ESLNQPHDLFYIIYTSGTTGQPKGVMLEHRNMANLMHFTFDQTNIAFHEKVLQYTTCSFDVCY<br>QEIFSTLLSGGQLYLITNELRRHVEKLFAFIQEKQISILSLPVSFLKFIFNEQDYAQSFPRSVKHII<br>TAGEQLVVTHELQKYLRQHRVFLHNHYGPSETHVVTTATMDPGQAIPELPPIGKPISNTGIYIL<br>DEGLQLKPEGIVGELYISGANVGRGYLHQPELTAEKFLDNPYQPGERMYRTGDLARWLPDG<br>QLEFLGRIDHQVKIRGHRIELGEIESRLLNHPAIKEAVVIDRADETGGKFLAAYVVLQKALSDEE<br>MRAYLAQALPEYMIPSFFVTLERIPVTPNGKTDRRALPKPEGSAKTKADYVAPTTELEQKLVAI<br>WEQILGVSPIGIQDHFFTLGGHSLKAIQLISRIQKEAQADVPLRVLFEQPTIQALAAYVEGSGVC<br>TEDTDSVDADTIIKTNYGEMTIENLFKSCSIKGPSWAIDDQEFTIYDQIQILTYDPKTNEEIYRPF<br>EYVYRHKVSKPRWKIIDENGNEIILTNDHSVMIERDGKLIEAKPSEINPDTDILITIGERSHHHHH<br>H
**15P**	LCGC^C-SAY-TycB2-TE-H_6	MVEKLKIQKIEKLEDFDNEYVYDISVDKETPYFFGNNILVHNSAYGGGEESAYLAIPQAEPQAY<br>YPVSSAQKRMLILNQLDPHSTVYNLPVAMILEGTLDKARLEHAISNLVARHESLRTSFHTINGE<br>PVSRIHEQGHLPIVYLETAEEQVNEVILGFMQPFDLVTAPLCRVGLVKLAENRHVLIIDMHHIISD<br>GVSSQLILNDFSRLYQNKALPEQRIHYKDFAVWEKAWTQTTDYQKQEKYWLDRFAGEIPVLN<br>LPMDYPRPAVQSFEGERYLFRTEKQLLESLQDVAQKTGTTLYMVLLAAYHVLLSKYSGQDDV<br>MIGTVTAGRVHPDTESMTGMFVNTLAMRNQSAPTKTFRQFLLEVKDNTLAAFEHGQYPFEEL<br>VEKLAIQRNRSRNPLFDTLFILQNMDADLIELDGLTVTPYVPEGEVAKFDLSLEASENQAGLSF<br>CFEFCTKLFARETIERMSLHYLQILQAVSANTEQELAQIEMLTAHEKQELLVHFNDTAALYPAE<br>STLSQLFEDQAQKTPEQTAVVFGDKRLTYRELNERANQLAHTLRAKGVQAEQSVGIMAQRSL<br>EMAIGIIAILKAGGAYVPIDPDYPNERIAYMLEDCEARLVLTQQQLAEKMTANVECLYLDEEGSY<br>SPQTENIEPIHTAADLAYIIYTSGTTGRPKGVMVEHRGIVNSVTWNRDEFALSVRDSGTLSLSF<br>AFDAFALTFFTLIVSGSTVVLMPDHEAKDPIALRNLIAAWECSYVVFVPSMFQAILECSTPADIR<br>SIQAVMLGGEKLSPKLVQLCKAMHPQMSVMNAYGPTESSVMATYLRDTQPDQPITIGRPIANT<br>AIYIVDQHHQLLPVGVVGEICIGGHGLARGYWKKPELTAEKFVANPAVPGERMYKTGDLGRW<br>LHDGTIDFIGRVDDQIKVRGYRIEVGEIEAVLLAYDQTNEAIVVAYQDDRGDSYLAAYVTGKTAI<br>EESELRAHLLRELPAYMVPTYLIQLDAFPLTPNGKVDRKALPKPEGKPATGAAYVAPATEVEA<br>KLVAIWENALGISGVGVLDHFFELGGHSLKAMTVVAQVHREFQIDLLLKQFFAAPTIRDLARLIE<br>HGSHKRFESRYGTAILLNQETARNVFCFTPIGAQSVYYQKLAAEIQGVSLYSFDFIQDDNRME<br>QYIAAITAIDPSGPYTLMGYSSGGNLAFEVAKELEERGYGVTDIILFDSYWKDKAIERTVAETEN<br>DIAQLFAEIGENTEMFNMTQEDFQLYAANEFVKQSFVRKTVSYVMFHNNLVNTGMTTAAIHLI<br>QSELEADEEAPVAAKWNESAWANATQRLLTYSGHGIHSRMLAGDYASQNASILQNILQELFIL<br>KRSRSHHHHHH

Table S3 List of sequences of recombinantly produced proteins (continued).*

Protein	Construct	Sequence
**16P**	SBP-TycA	MDEKTTGWRGGHVVEGLAGELEQLRARLEHHPQGQREPMVANQANLIDNKRELEQHALVP<br>YAQGKSIHQLFEEQAEAFPDRVAIVFENRRLSYQELNRKANQLARALLEKGVQTDSIVGVMME<br>KSIENVIAILAVLKAGGAYVPIDIEYPRDRIQYILQDSQTKIVLTQKSVSQLVHDVGYSGEVVVLD<br>EEQLDARETANLHQPSKPTDLAYVIYTSGTTGKPKGTMLEHKGIANLQSFFQNSFGVTEQDRI<br>GLFASMSFDASVWEMFMALLSGASLYILSKQTIHDFAAFEHYLSENELTIITLPPTYLTHLTPERI<br>TSLRIMITAGSASSAPLVNKWKDKLRYINAYGPTETSICATIWEAPSNQLSVQSVPIGKPIQNTH<br>IYIVNEDLQLLPTGSEGELCIGGVGLARGYWNRPDLTAEKFVDNPFVPGEKMYRTGDLAKWL<br>TDGTIEFLGRIDHQVKIRGHRIELGEIESVLLAHEHITEAVVIAREDQHAGQYLCAYYISQQEATP<br>AQLRDYAAQKLPAYMLPSYFVKLDKMPLTPNDKIDRKALPEPDLTANQSQAAYHPPRTETESI<br>LVSIWQNVLGIEKIGIRDNFYSLGGDSIQAIQVVARLHSYQLKLETKDLLNYPTIEQVALFVKSTT<br>RKSDQGIIAGNVPLTPIQKWFFGKNFTNTGHWNQSSVLYRPEGFDPKVIQSVMDKIIEHHDAL<br>RMVYQHENGNVVQHNRGLGGQLYDFFSYNLTAQPDVQQAIEAETQRLHSSMNLQEGPLVKV<br>ALFQTLHGDHLFLAIHHLVVDGISWRILFEDLATGYAQALAGQAISLPEKTDSFQSWSQWLQE<br>YANEADLLSEIPYWESLESQAKNVSLPKDYEVTDCKQKSVRNMRIRLHPEETEQLLKHANQA<br>YQTEINDLLLAALGLAFAEWSKLAQIVIHLEGHGREDIIEQANVARTVGWFTSQYPVLLDLKQT<br>APLSDYIKLTKENMRKIPRKGIGYDILKHVTLPENRGSLSFRVQPEVTFNYLGQFDADMRTELF<br>TRSPYSGGNTLGADGKNNLSPESEVYTALNITGLIEGGELVLTFSYSSEQYREESIQQLSQSY<br>QKHLLAIIAHCTEKKEVERTPSDFSVKGLQMEEMDDIFELLANTLR
**17P**	TycB1-TycB2-TE-H_6	MSVFSKEQVQDMYALTPMQEGMLFHALLDQEHNSHLVQMSISLQGDLDVGLFTDSLHVLVE<br>RYDVFRTLFLYEKLKQPLQVVLKQRPIPIEFYDLSACDESEKQLRYTQYKRADQERTFHLAKD<br>PLMRVALFQMSQHDYQVIWSFHHILMDGWCFSIIFDDLLAIYLSLQNKTALSLEPVQPYSRFIN<br>WLEKQNKQAALNYWSDYLEAYEQKTTLPKKEAAFAKAFQPTQYRFSLNRTLTKQLGTIASQN<br>QVTLSTVIQTIWGVLLQKYNAAHDVLFGSIVSGRPTDIVGIDKMVGLFINTIPFRVQAKAGQTFS<br>ELLQAVHKRTLQSQPYEHVPLYDIQTQSVLKQELIDHLLVIENYPLVEALQKKALNQQIGFTITA<br>VEMFEPTNYDLTVMVMPKEELAFRFDYNAALFDEQVVQKLAGHLQQIADCVANNSGVELCQI<br>PLLTEAETSQLLAKRTETAADYPAATMHELFSRQAEKTPEQVAVVFADQHLTYRELDEKSNQL<br>ARFLRKKGIGTGSLVGTLLDRSLDMIVGILGVLKAGGAFVPIDPELPAERIAYMLTHSRVPLVVT<br>QNHLRAKVTTPTETIDINTAVIGEESRAPIESLNQPHDLFYIIYTSGTTGQPKGVMLEHRNMANL<br>MHFTFDQTNIAFHEKVLQYTTCSFDVCYQEIFSTLLSGGQLYLITNELRRHVEKLFAFIQEKQISI<br>LSLPVSFLKFIFNEQDYAQSFPRCVKHIITAGEQLVVTHELQKYLRQHRVFLHNHYGPSETHVV<br>TTCTMDPGQAIPELPPIGKPISNTGIYILDEGLQLKPEGIVGELYISGANVGRGYLHQPELTAEK<br>FLDNPYQPGERMYRTGDLARWLPDGQLEFLGRIDHQVKIRGHRIELGEIESRLLNHPAIKEAV<br>VIDRADETGGKFLCAYVVLQKALSDEEMRAYLAQALPEYMIPSFFVTLERIPVTPNGKTDRRAL<br>PKPEGSAKTKADYVAPTTELEQKLVAIWEQILGVSPIGIQDHFFTLGGHSLKAIQLISRIQKECQ<br>ADVPLRVLFEQPTIQALAAYVEGGEESAYLAIPQAEPQAYYPVSSAQKRMLILNQLDPHSTVY<br>NLPVAMILEGTLDKARLEHAISNLVARHESLRTSFHTINGEPVSRIHEQGHLPIVYLETAEEQVN<br>EVILGFMQPFDLVTAPLCRVGLVKLAENRHVLIIDMHHIISDGVSSQLILNDFSRLYQNKALPEQ<br>RIHYKDFAVWEKAWTQTTDYQKQEKYWLDRFAGEIPVLNLPMDYPRPAVQSFEGERYLFRT<br>EKQLLESLQDVAQKTGTTLYMVLLAAYHVLLSKYSGQDDVMIGTVTAGRVHPDTESMTGMFV<br>NTLAMRNQSAPTKTFRQFLLEVKDNTLAAFEHGQYPFEELVEKLAIQRNRSRNPLFDTLFILQN<br>MDADLIELDGLTVTPYVPEGEVAKFDLSLEASENQAGLSFCFEFCTKLFARETIERMSLHYLQI<br>LQAVSANTEQELAQIEMLTAHEKQELLVHFNDTAALYPAESTLSQLFEDQAQKTPEQTAVVFG<br>DKRLTYRELNERANQLAHTLRAKGVQAEQSVGIMAQRSLEMAIGIIAILKAGGAYVPIDPDYPN<br>ERIAYMLEDCRRLVLTQQQLAEKMTANVECLYLDEEGSYSPQTENIEPIHTAADLAYIIYTSGTT<br>GRPKGVMVEHRGIVNSVTWNRDEFALSVRDSGTLSLSFAFDAFALTFFTLIVSGSTVVLMPDH<br>EAKDPIALRNLIAAWECSYVVFVPSMFQAILECSTPADIRSIQAVMLGGEKLSPKLVQLCKAMH<br>PQMSVMNAYGPTESSVMATYLRDTQPDQPITIGRPIANTAIYIVDQHHQLLPVGVVGEICIGGH<br>GLARGYWKKPELTAEKFVANPAVPGERMYKTGDLGRWLHDGTIDFIGRVDDQIKVRGYRIEV<br>GEIEAVLLAYDQTNEAIVVAYQDDRGDSYLAAYVTGKTAIEESELRAHLLRELPAYMVPTYLIQL<br>DAFPLTPNGKVDRKALPKPEGKPATGAAYVAPATEVEAKLVAIWENALGISGVGVLDHFFELG<br>GHSLKAMTVVAQVHREFQIDLLLKQFFAAPTIRDLARLIEGSHKRFESRYGTAILLNQETARNV<br>FCFTPIGAQSVYYQKLAAEIQGVSLYSFDFIQDDNRMEQYIAAITAIDPSGPYTLMGYSSGGNL<br>AFEVAKELEERGYGVTDIILFDSYWKDKAIERTVAETENDIAQLFAEIGENTEMFNMTQEDFQL<br>YAANEFVKQSFVRKTVSYVMFHNNLVNTGMTTAAIHLIQSELEADEEAPVAAKWNESAWANA<br>TQRLLTYSGHGIHSRMLAGDYASQNASILQNILQELFILKRSHHHHHH
**18P**	H_6-Smt3-Aes^{C}-<br>SVYGTSCSTGDTD-<br>$LCGC^{N}$-SBP	MGSSHHHHHHSSGLVPRGSHMASMSDSEVNQEAKPEVKPEVKPETHINLKVSDGSSEIFFKI<br>KKTTPLRRLMEAFAKRQGKEMDSLRFLYDGIRIQADQTPEDLDMEDNDIIEAHREQIGGSRIEF<br>IEFEIEDLGVMEIDVYDIEVDGNHNFFGNDILVHNSVYGTSCSTGDTDSVDADTIIKTNYGEMTI<br>ENLFKSSSIKGPSWAIDDQEFTIYDQIQILTYDPKTNEEIYRPFEYVYRHKVSKPRWKIIDENGN<br>EIILTNDHSVMIERDGKLIEAKPSEINPDTDILITIGERSMDEKTTGWRGGHVVEGLAGELEQLR<br>ARLEHHPQGQREP

*Intein fragments are marked in blue (Int^{N}: light blue, Int^{C}: dark blue). The introduction of native extein residues is marked in red. NRPS are marked in dark green and the thioesterase domain is marked in violet. MBP is marked in orange, superfolder GFP in light green, diSUMO in magenta, and thioredoxin in yellow.

Table S4 List of the split intein nucleotide sequences used in the respective expression plasmids.*

**N-terminal Intein Fragment**	
Intein	Nucleotide Sequence
CP21 PolB1 (used in constuct **4P**)	AGCGTTGTTGGCGATAGCATTATCAAAGTGAATGGCAAAAACATCAAAATCGAGGACTTCTA<br>TGATAGCATCAAAGTTGATCCGATTGTGACCAAAAGCGGCAATAATGTTAAACTGGTGGATA<br>AC**TGT**TTTACCGAAAGCGTGAATAAAAACCTGCAGATCGAAACCAAAAAAATCAACTATATCA<br>TGAAACACAAAGTGAAAAAAGAGTTCTTCAAAATCAAAGTCAACAACAAAGAAGTGGTGGTC<br>ACCGAAGATCATTCAATTATGGTTCTGCGTAACAGCGAACTGATTGAAGTTAAACCGCGTGA<br>TATCAAAAACGGCGATCTGATTATTCTGAATGAT
LCGC14 PolB2 (used in construct **5P**)	AGCGTGGACGCTGATACAATTATTAAGACGAACTACGGAGAGATGACTATCGAAAATTTGTT<br>TAAGTCT**TGT**AGCATTAAAGGTCCGTCCTGGGCAATCGACGATCAGGAATTTACCATTTATG<br>ACCAGATCCAAATTTTGACTTACGACCCAAAGACGAACGAGGAAATTTACCGTCCCTTCGAA<br>TATGTATATCGTCATAAGGTCTCTAAGCCTCGCTGGAAAATCATCGACGAAAATGGGAACGA<br>GATTATTTTAACGAACGACCACTCCGTTATGATTGAGCGCGATGGTAAACTTATTGAGGCGA<br>AGCCGAGCGAAATCAACCCGGACACGGATATTCTGATTACTATTGGTGAG
GOM-OPS-1 PolB2 (used in construct **6P**)	AGCGTAGCGTTCAATTCCATCATCGAGATCGATGGCATCAAGGACACTATCGAATCGTGGTT<br>TAATAAATTGGCTGAAGAACATGGTCGCCACGTAGATGGCGAAAAAGAGTTCACAAAAATTT<br>CCTCCCTGGACCTGCACACTCCAACATACGATGTGGCTTATGAC**TGT**ATGGTAGACAAGCC<br>TCTGATGACCATTTATCGCCATAAAATTGAAAAGAAGATGTATACTGTGACATCTGTAGACG<br>GACATAGCGTTACTACGACTGCCGACCATAGCCTGATGGTAATGCGCGGGGGAAACATTAT<br>TGAGATCATCCCAACGGACATTTTATCTGGAGATCAACTTGTAATCTTCGAG
**C-terminal Intein Fragment**	
Intein	Nucleotide Sequence
CP21 PolB1 (used in constuct **7P**)	ATGATCGTGACCGAAAACTTTCAGGTTGAAAGCCTGGGTATTCAAGAACTGGATGTGTATGA<br>TATTGAGGTGGATAGCAACCACAACTTTTTTGCCAATGATATCCTGGTGCATAAC
LCGC14 PolB2 (used in construct **8P** and **15P**)	ATGGTGGAGAAATTGAAGATTCAAAAGATTGAGAAACTGGAAGACTTTGACAACGAGTATGT<br>CTATGACATCTCCGTGGATAAAGAGACGCCATATTTCTTCGGCAATAACATCCTTGTTCATAA<br>C
GOM-OPS-1 PolB2 (used in construct **9P**)	ATGCATGAACGCAAGTACAAGCTGGTCGATATTGCCAAGGTCGAGGAGGTAAAATATACTG<br>ATGAATACGTTTATGACGTTGTCATGTTCGAGCCAGAAAGTCCGTACTTCGTTGCAAACGAT<br>ATTTTAGTCCATAAT

*Note that the nucleotides highlighted in bold red denote the mutation sites corresponding to Cys to Ser substitutions. In CP21 PolB1, the codon was altered to TCT to generate construct **1P**, in LCGC14 PolB2, the codon was altered to AGT to generate constructs **2P** and **14P**, and in GOM-OPS-1 PolB2, the codon was altered to AGC to generate construct **3P**.

Supplementary Figures

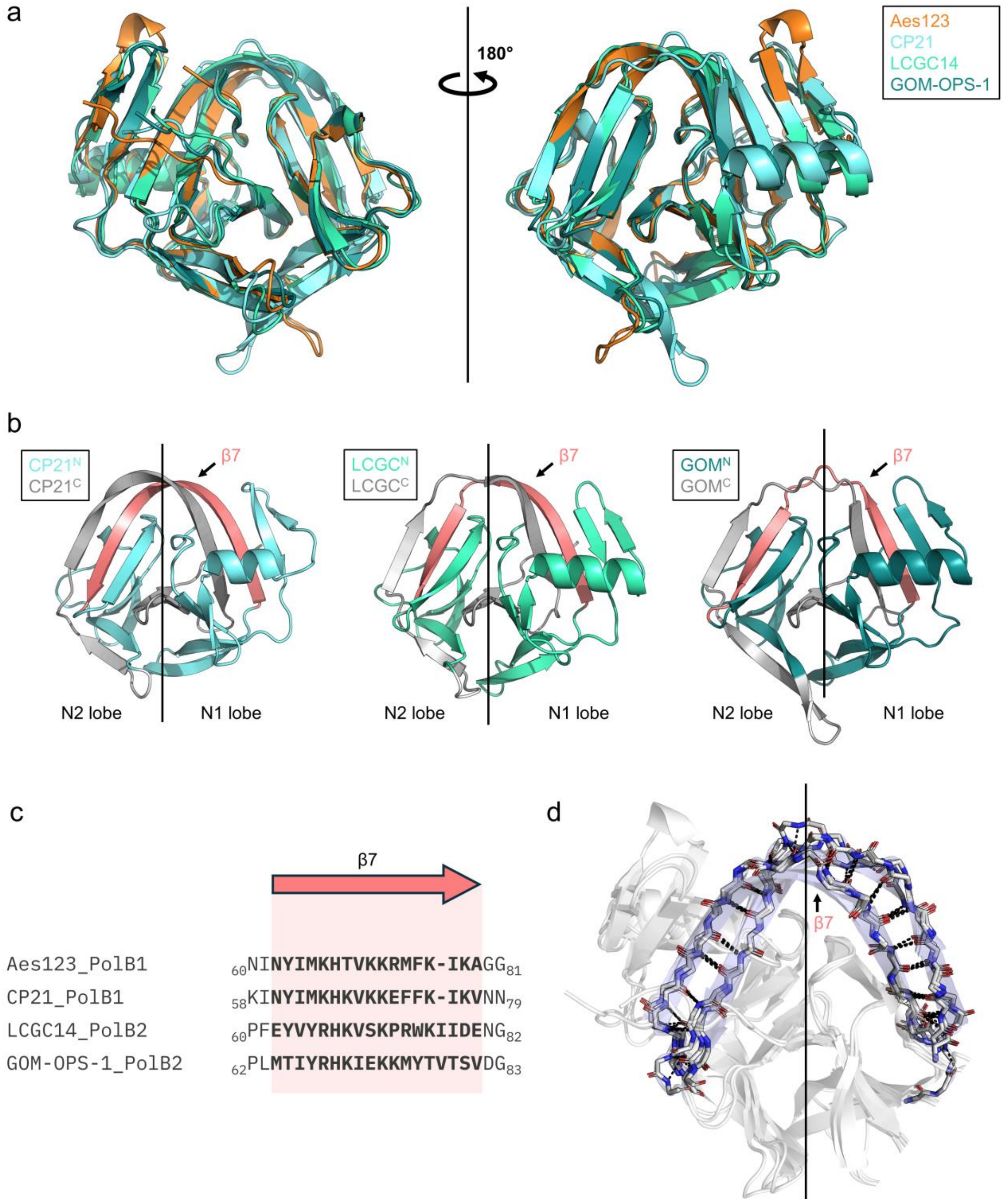

Figure S1 Structural prediction of the three investigated cysteine-less split inteins. (a) AlphaFold3 structural models of the CP21, LCGC14 and GOM-OPS-1 split inteins aligned to the experimentally determined crystal structure of the Aes123 PolB1 split intein (PDB ID: 9HTH).[4] (b) AlphaFold3 models of the three new split inteins. The Int^{N} fragments are marked in color while the Int^{C} fragments are marked in gray as indicated. The black line highlights the conserved pseudo two-fold symmetric axis of the usual Hedgehog/Intein (HINT) fold.[10] The β-strand β7 is marked in red and was used to define the border between the N1 and N2 lobes (but was counted to the N2 lobe in full length) for the computational aggregation analysis. (c) Sequence sections of the structural alignment shown in (a) based on the AlphaFold3 models encompassing the β-strand β7 of the Int^{N} fragments. (d) Structural overlay shown in (a) of all four inteins highlighting the β-strand β7/β13-mediated interaction between the Int^{N} and Int^{C} fragments.

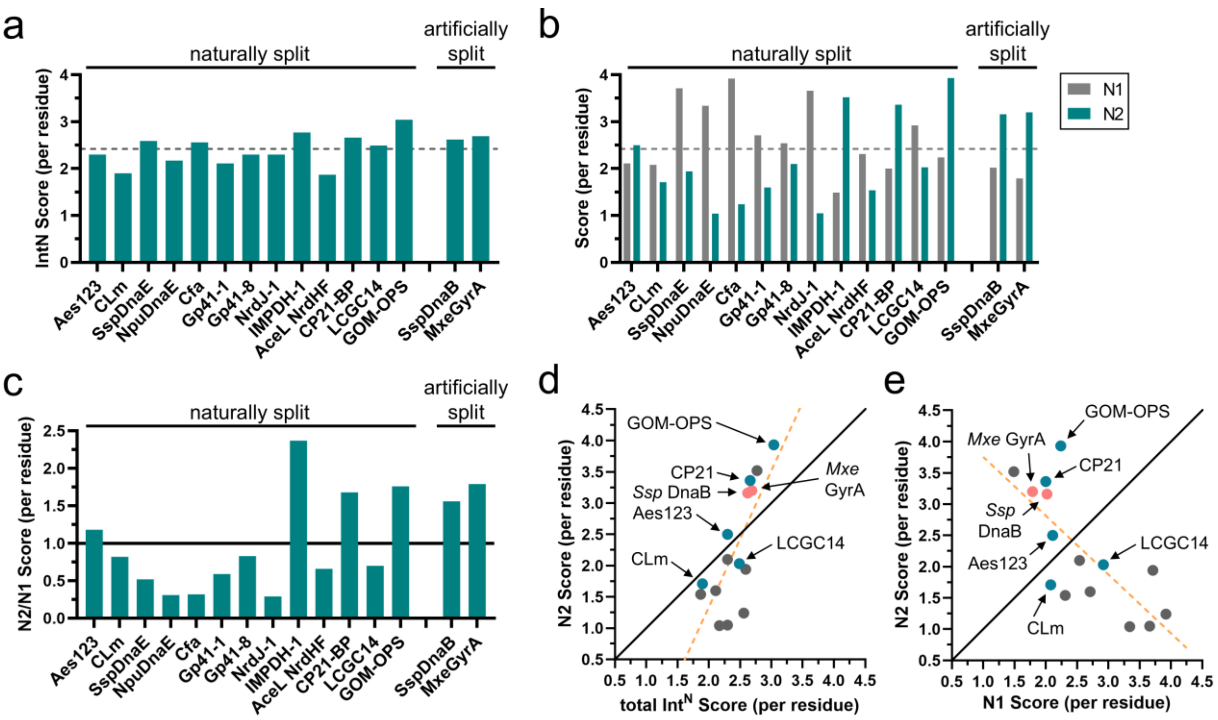

f

Intein	N1 Score	N2 Score	N_{total} Score	N2/N1 Score
Aes123 PolB1	2.11	2.50	2.30	1.18
CLm	2.08	1.71	1.90	0.82
Ssp DnaE	3.71	1.94	2.59	0.52
Npu DnaE	3.34	1.04	2.17	0.31
Cfa	3.92	1.24	2.56	0.32
Gp41-1	2.71	1.60	2.11	0.59
Gp41-8	2.54	2.10	2.30	0.83
NrdJ-1	3.66	1.05	2.30	0.29
IMPDH-1	1.49	3.52	2.77	2.37
AceL NrdHF	2.31	1.54	1.87	0.66
CP21-BP	2.00	3.36	2.66	1.68
LCGC14	2.92	2.03	2.49	0.70
GOM-OPS	2.24	3.93	3.04	1.76
CP21(C43S)	1.73	3.36	2.52	1.94
LCGC(C24S)	2.81	2.03	2.43	0.72
GOM(C57S)	1.65	3.93	2.73	2.38
Ssp DnaB	2.02	3.16	2.62	1.56
Mxe GyrA	1.79	3.20	2.69	1.79

Figure S2 Sequence-based aggregate prediction of commonly used native and artificially split inteins. The artificially split inteins are *Ssp* DnaB[11] and *Mxe* GyrA.[12] (a) Int^N score normalized to the fragment sequence length. The dotted line marks the average score. (b) Scores of the N1 and N2 lobes normalized to the fragment sequence length. The border between the N1 and N2 lobes is defined by β-strand β7 and the dotted line marks the average Int^N score shown in (a). (c) Ratio of the N2/N1 scores normalized to the sequence length. (d/e) Plots of the N2 scores per residue against the overall IntN score (d) and against the N1 scores per residue (e). This analysis includes some commonly used natively split inteins (as shown in Figure 2e, f) next to the plots of the artificially split *Ssp* DnaB[11] and *Mxe* GyrA[12] inteins marked as red circles. Solid black lines indicate the theoretical line of a 1:1 positive correlation and dashed red lines represent the linear regression fit of the experimental data. (f) Table presenting the calculated values of the N1, N2, N_{total}, and the N2/N1 scores for a set of commonly used natively split and artificially split inteins.

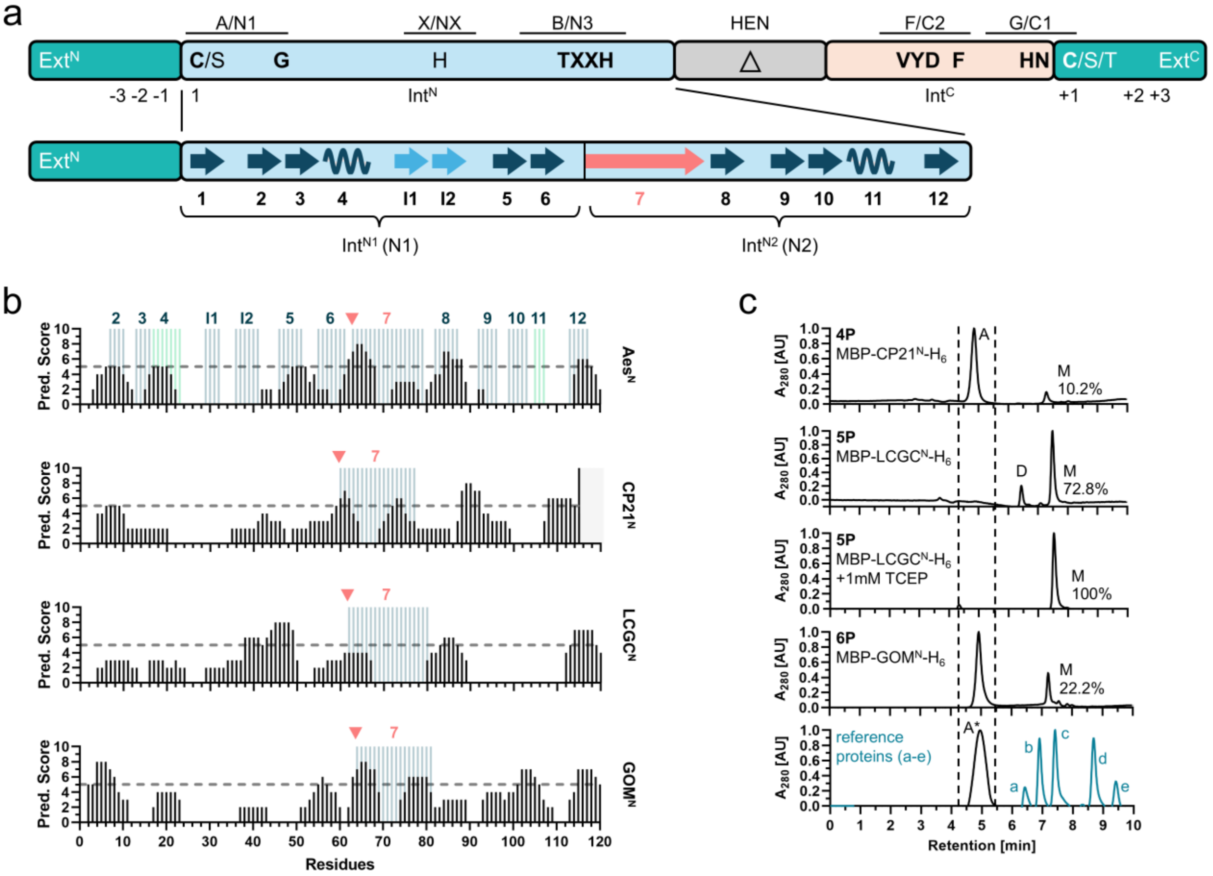

Figure S3 Computational prediction and experimental verification of Int^N precursor aggregation. Aggregation-prone sites were computationally predicted within the native Int^N fragments containing the non-essential cysteines of the three new split inteins. The same Int^N fragments were used for the experimental investigation of size-exclusion chromatography (SEC). (a) Illustration of conserved sequence motifs and secondary structure elements in the minimal intein horseshoe fold of *cis*-inteins (top) and the Int^N fragment of split inteins (bottom). Additionally, one optional insertion of β-strands I1 & I2 is shown that is often found in cysteine-less inteins.[4, 13] The border between N1 and N2 lobes was defined at the beginning of β-strand β7 as used previously[4] and in contrast to a symmetry axis within β-strand β7 described elsewhere.[14] (b) Consensus predictions of amyloidogenic pattern formation using AMYLPRED2[5] of the native Int^N fragments of the three investigated cysteine-less split inteins compared to the Aes123 PolB1 split intein.[4] Secondary structure elements are marked in blue (β-sheets) and green (α-helices). The red arrowheads indicate the defined border between the N1 and N2 lobes. (c) SEC UV-profiles of the investigated constructs **4P**, **5P** and **6P** to analyze the appearance of the aggregated (A), dimeric (D) and monomeric (M) species compared to standard globular protein markers used as reference (bottom panel). The constructs were adjusted to a concentration of 10 µM and incubated for 24 h at 15 °C prior to SEC analysis to exclude concentration-dependent artefacts in the aggregate proportion. As a reference for the aggregated species (A*) the previously investigated aggregate of MBP-Aes^N-H6 was used.[4] Note that the dimeric species of **5P** disappears upon addition of TCEP as reducing agent, suggesting it was caused by disulfide bond.

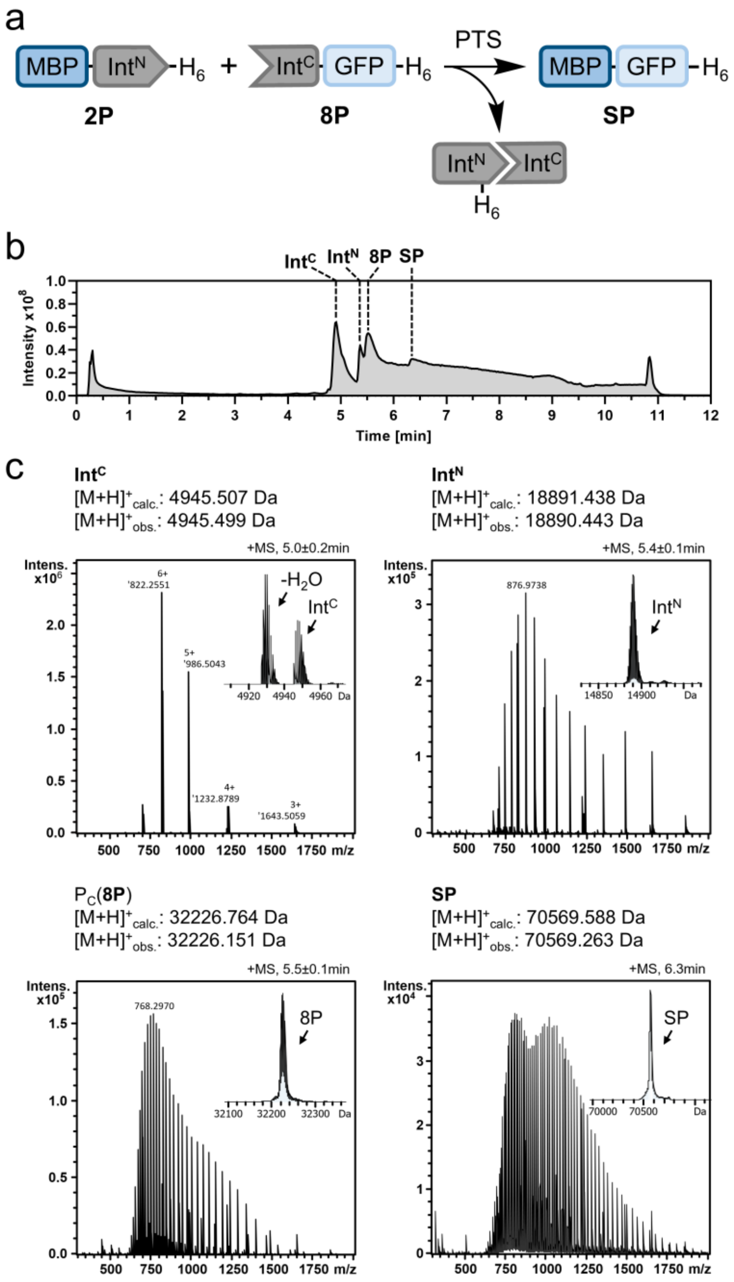

Figure S4 LC-MS analysis of the PTS reaction of the LCGC14 precursors. Additional data to the PTS reaction analyzed in the Figure 4b (middle panel) (a) Schematic illustration of the PTS reaction. (b) LC-MS analysis of the total PTS reaction. Shown is a total ion chromatogram after HPLC separation. Identifiable proteins are indicated. (c) MS spectra corresponding to the selected HPLC fractions as indicated in (b), with insets displaying the deconvoluted masses. The loss of one water molecule observed for the Int^C protein can be explained with the succinimide form of this species.

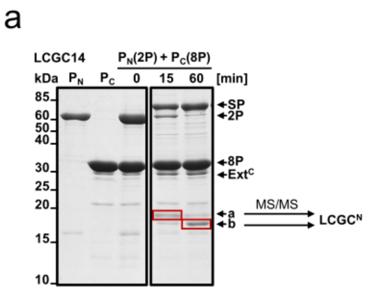

b

>LCGC^N_post-splicing(band_a)

SVDADTIIKT NYGEMTIENL FKSSSIKGPS WAIDDQEFTI YDQIQILTYD PKTNEEIYRP

FEYVYRHKVS KPRWKIIDEN GNEIILTNDH SVMIERDGKL IEAKPSEINP DTDILITIGE

GSHHHHHH

>LCGC^N_post-splicing(band_b)

SVDADTIIKT NYGEMTIENL FKSSSIKGPS WAIDDQEFTI YDQIQILTYD PKTNEEIYRP

FEYVYRHKVS KPRWKIIDEN GNEIILTNDH SVMIERDGKL IEAKPSEINP DTDILITIGE

GSHHHHHH

Figure S5 Analysis of the unknown double band in Figure 4b (marked with a hashtag (#)). This analysis was performed to clarify the nature of the two bands appearing in the size range expected for the excised Int^N fragment of the LCGC14 intein. (a) Coomassie-stained SDS-PAGE gel analysis of the PTS reaction using precursor MBP-$LCGC^N$(C24S)-H_6 (**2P**) and $LCGC^C$-GFP-H_6 (**8P**), similar to the data shown in Figure 4b. The red rectangles indicate the two bands excised from the gel and analyzed by LC-MS/MS following tryptic digest (a: upper band, b: lower band). (b) Results of the LC-MS/MS analyses of bands a and b. The observed 94.5% sequence coverage (underlined sequences) from both digests (a and b) matches both protein bands to the free $LCGC^N$ fragment post-splicing, suggesting that they are identical species in terms of primary sequence and migrate differently on the gel for unknown reasons. Note that the LC-MS/MS analysis indicates no cleavage events or post-translational modification that could account for the observed difference in electrophoretic mobility between the two bands. Uncropped SDS-PAGE images are shown in Figure S13.

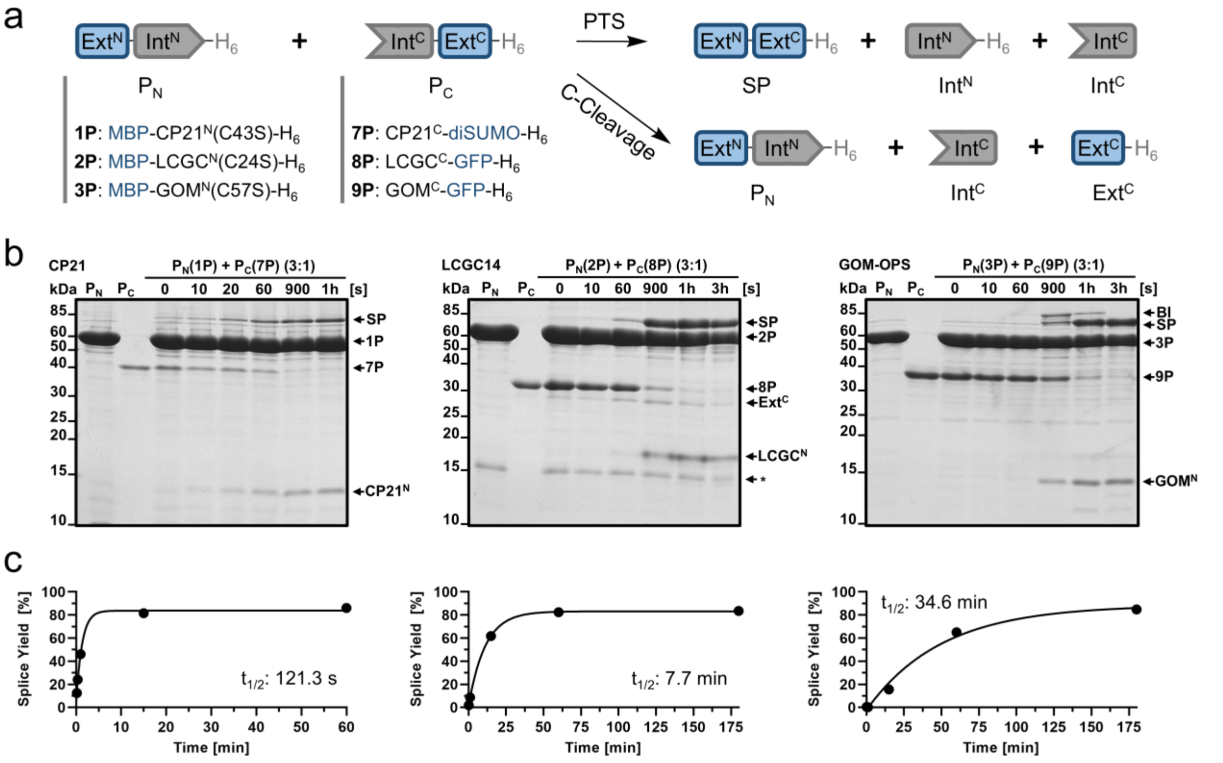

Figure S6 Splice assays with Int^N precursor (P_N) in excess to analyze the Int^C precursor (P_C) activity. (a) Scheme of the PTS reaction with C-cleavage as potential side reaction. (b) SDS-PAGE analysis of the PTS reactions illustrated in (a) at 37 °C using 30 µM of P_N and 10 µM of P_C, and in the absence of any reducing agents. These experiments were repeated two times. Shown are Coomassie-stained gels. (c) Splice product formation relative to the P_C concentration calculated by densitometric analysis plotted against time to extract the splice kinetics by fitting to a one-phase exponential equation. Note that the MWs of the excised Int^C fragments are too small to be visible on these gels. Note that under these conditions no extra band for the excised $LCGC^N$ fragment was observed (compare Figures S4 and S5). MWs are shown in Table S1. (*) denotes protein contaminations. Uncropped SDS-PAGE images are shown in Figure S13.

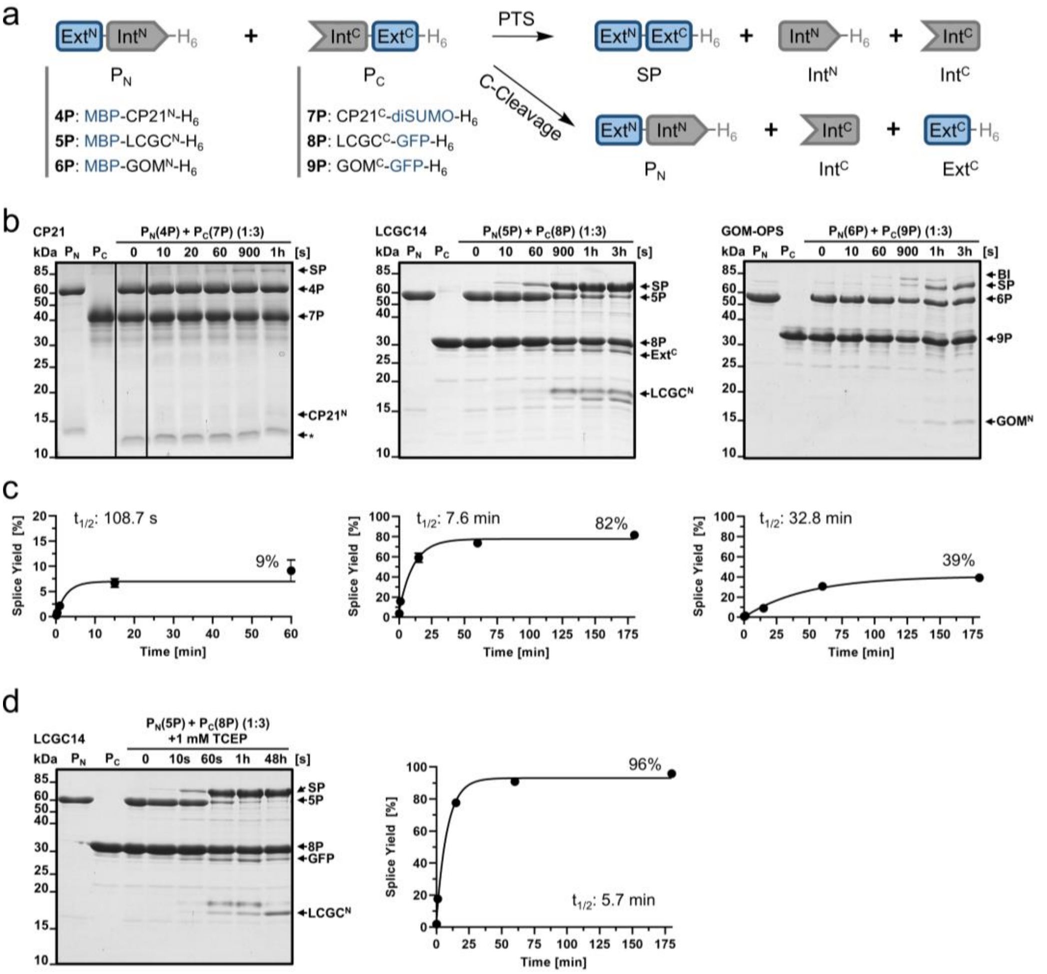

Figure S7 Splice activity of the native cysteine-independent split inteins without prior SEC purification. The Int^{N} precursors (P_N) used in these experiments each contained one native cysteine at a non-conserved position. (a) Scheme of the PTS reaction with C-cleavage as potential side reaction. (b) SDS-PAGE analysis of the PTS reactions illustrated in (a) at 37 °C using 10 µM of P_N and 30 µM of P_C, and in the absence of any reducing agents. These experiments were repeated two times. Shown are Coomassie-stained gels. (c) Splice product formation calculated by densitometric analysis plotted against time to extract the splice kinetics by fitting to a one-phase exponential equation. (d) SDS-PAGE analysis of the PTS reaction at 37 °C using 10 µM of **8P** and 30 µM of **4P** in presence of 1 mM TCEP (left panel) and splice product formation calculated by densitometric analysis plotted against time to extract the splice kinetics by fitting to a one-phase exponential equation (right panel). Note that the MWs of the excised Int^{C} fragments are too small to be visible on these gels. MWs are shown in Table S1. (*) denotes protein contaminations. Uncropped SDS-PAGE images are shown in Figure S13.

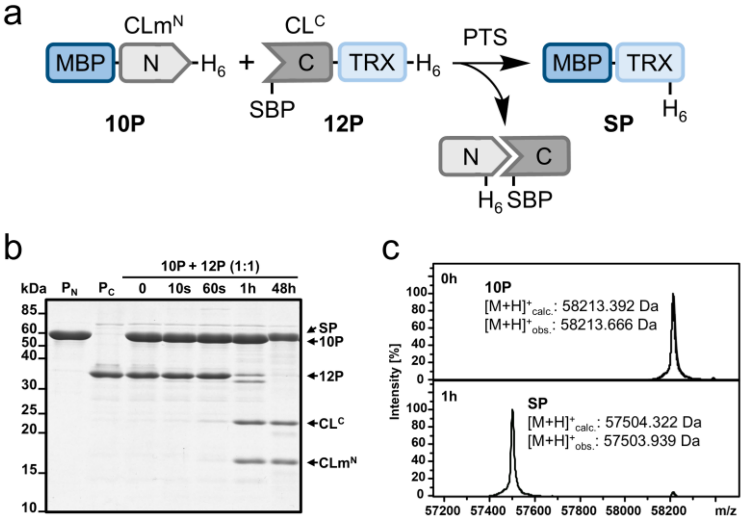

Figure S8 Cross reactivity between the cysteine-less CLm and CL inteins. (a) Schematic illustration of the PTS reaction used to assess the cross reactivity between the CLm and CL inteins. (b) Coomassie-stained SDS-PAGE analysis of the PTS reaction performed with equimolar concentrations (5 µM) of precursor MBP-CLm^N-H_6 (**10P**) and SBP-CL^C-TRX-H_6 (**12P**) at 37 °C. The minor shift in the MW between **10P** and SP is due to the similar size of CLm^N and TRX. (c) LC-MS analysis of the PTS reaction confirming correct splice product formation. Calculated molecular masses are: **10P** = 58.2 kDa, **12P** = 33.8 kDa, SP = 57.5 kDa, CL^C = 19.9 kDa, and CLm^N = 14.7 kDa. Uncropped SDS-PAGE images are shown in Figure S13.

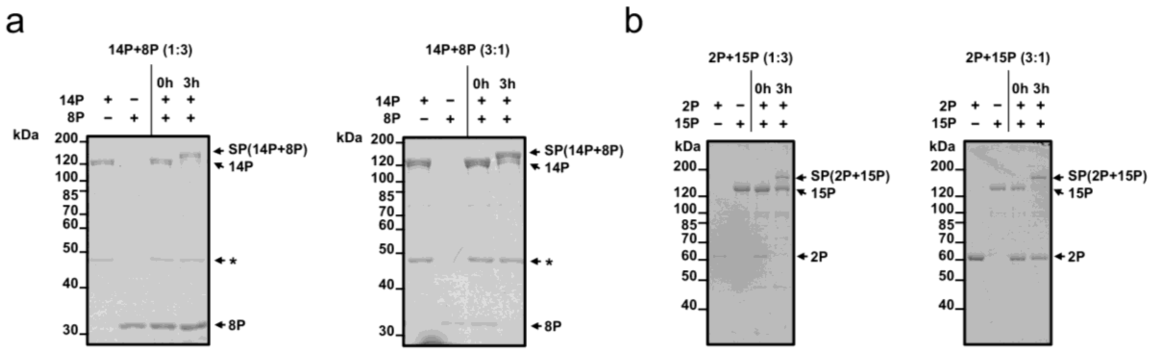

Figure S9 Analysis of PTS activity of LCGC intein precursors Aes^C-TycB1-LCGC^N (**14P**) and LCGC^C-TycB2-TE (**15P**). The purified LCGC intein precursors **14P** and **15P** were combined with MBP-LCGC^N (**2P**) and LCGC^C-GFP (**8P**), respectively. Providing **2P** and **8P** in 3-fold molar excess over their splice partners **14P** and **15P**, respectively, shows that both **14P** and **15P** can be virtually completely converted into the respective splice products (SP). Therefore, the incomplete PTS reaction of **14P** and **15P** shown in Figure 7b cannot be due to partial inactivity of one of the LCGC split intein fragments, but likely is a result of the two large exteins TycB1 and TycB2-TE. (*) denotes a protein contamination. Uncropped SDS-PAGE images are shown in Figure S13.

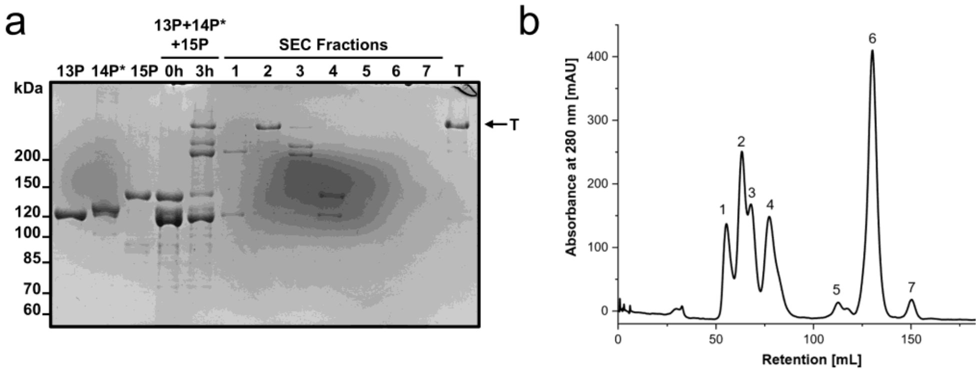

Figure S10 Purification of the three-segment splice product **13P-14P*-15P** (**T**) as shown in Figure 7 by size-exclusion chromatography (SEC). (a) Coomassie-stained SDS-PAGE gel of the precursor proteins, the tandem PTS reaction, and selected SEC elution fractions. (b) SEC chromatogram. Indicated 1 mL fractions were used for the SDS-PAGE analysis shown in (a). Fraction 2 contained the purified splice product. Note that fraction 1 contained precursor **13P** and splice product **13P-14P*** despite lower molecular weights of these proteins compared to the three-segment splice product (**T**). This finding indicates an apparent higher molecular weight caused by the TycA extein. The underlying partially oligomeric or aggregated species of these proteins might explain the partial inactivity of the precursor **13P** (TycA-CLm^{N}) in the PTS reaction. Uncropped SDS-PAGE image is shown in Figure S13.

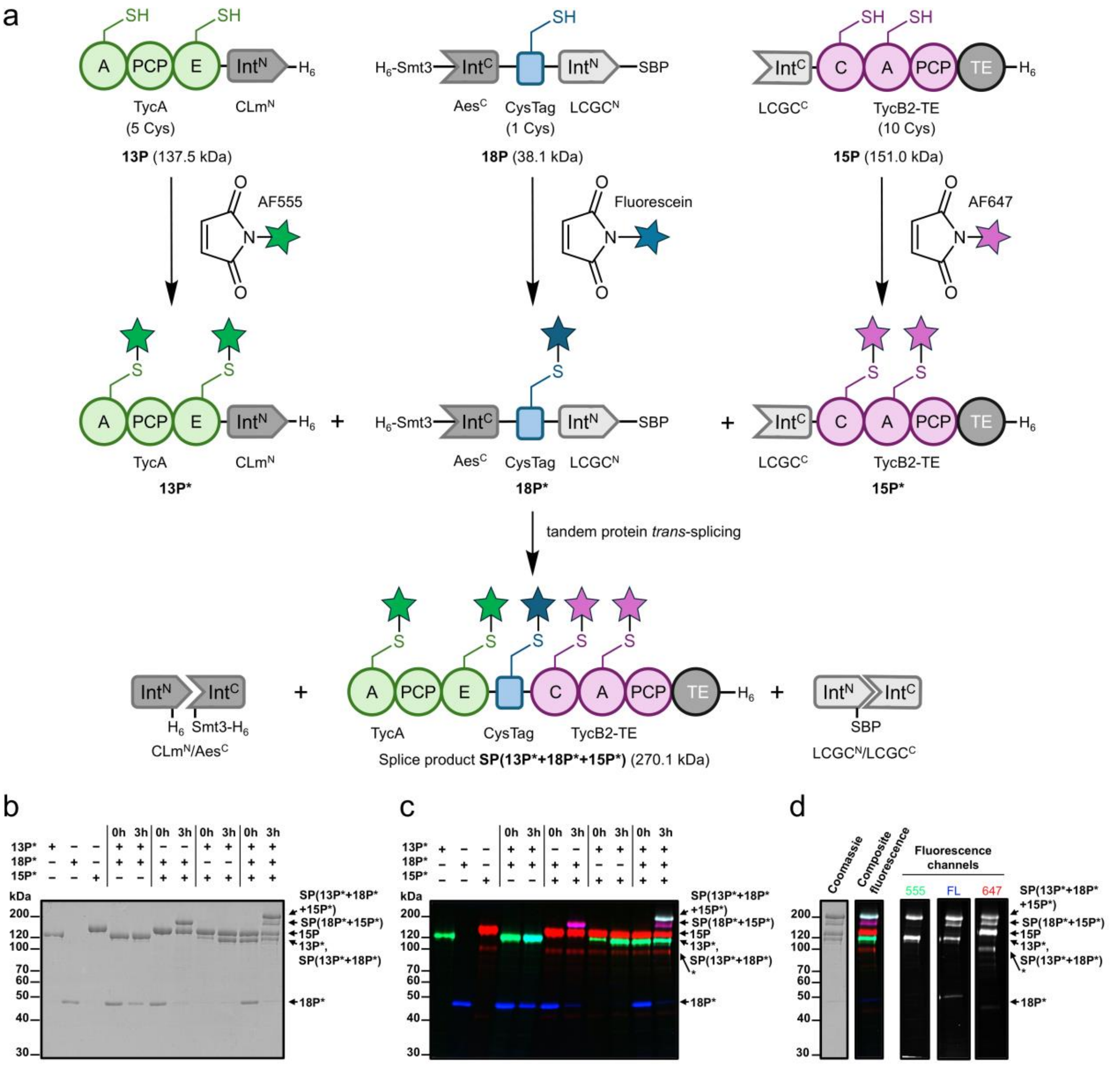

Figure S11. Three-segment protein labeling to generate a selectively labeled triple-color protein. (a) Reaction scheme. Note that the first segment (TycA) contains a total of 5 cysteines, the second segment (13 aa CysTag) contains 1 cysteine and the third segment (TycB2-TE) contains 10 cysteines. Individual thiol bioconjugation reactions were quenched with excess DTT prior to tandem PTS. (b) Analysis of the purified precursors, single and tandem PTS reactions using a Coomassie-stained SDS-PAGE gel. Precursor proteins were mixed at equimolar concentrations (4.5 µM) in the indicated combinations and incubated at 25 °C for 3 h. For the reaction **13P*** + **18P*** + **15P***, the precursor proteins **13P*** and **18P*** were pre-incubated for 15 min at 25 °C before adding **15P***. Note that the **13P*** precursor was added at too low concentration in the 0 h sample of the **13P*** + **15P*** reaction. (c) Fluorescence scan (composite fluorescence of the AF555, fluorescein and AF647 channels) of the gel shown in b). Note that the triple-color splice product (**13P+18P*+15P***) appears in white color. (d) Side-by-side comparison of the last lane with the triple-color splice product (**13P+18P*+15P***) shown in b) and c) for fluorescence analysis with the individual fluorophore channels (555: AlexaFluor 555, FL: Fluorescein, 647: AlexaFluor 647). (*) denotes a protein contamination of purified **15P***. Calculated molecular weights for the splice products of the single PTS reactions are **SP(13P*+18P*)** = 142.4 kDa and **SP(18P*+15P*)** = 165.8 kDa (without the masses of the fluorophores). Uncropped SDS-PAGE images are shown in Figure S13.

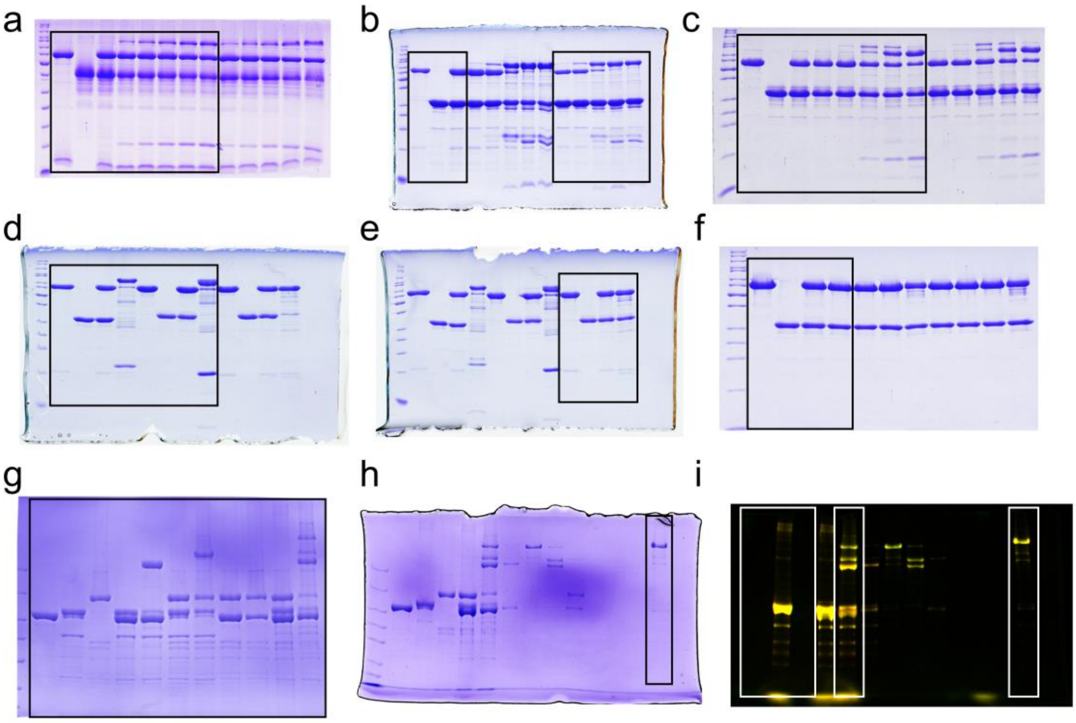

Figure S12 Unprocessed SDS-PAGE images of the figures shown in the main text. The black frame indicates the section used for the figures (a) Figure 4b (left panel), (b) Figure 4b (middle panel) (c) Figure 4b (right panel), (d) Figure 5b (first two panels), (e) Figure 5b (third panel from left), (f) Figure 5b (last panel from left), (g) Figure 7b (left panel), (h) Figure 7b (right panel), (i) Figure 7c. PageRuler™ unstained protein ladder (Thermo Scientific #26614) was used as marker.

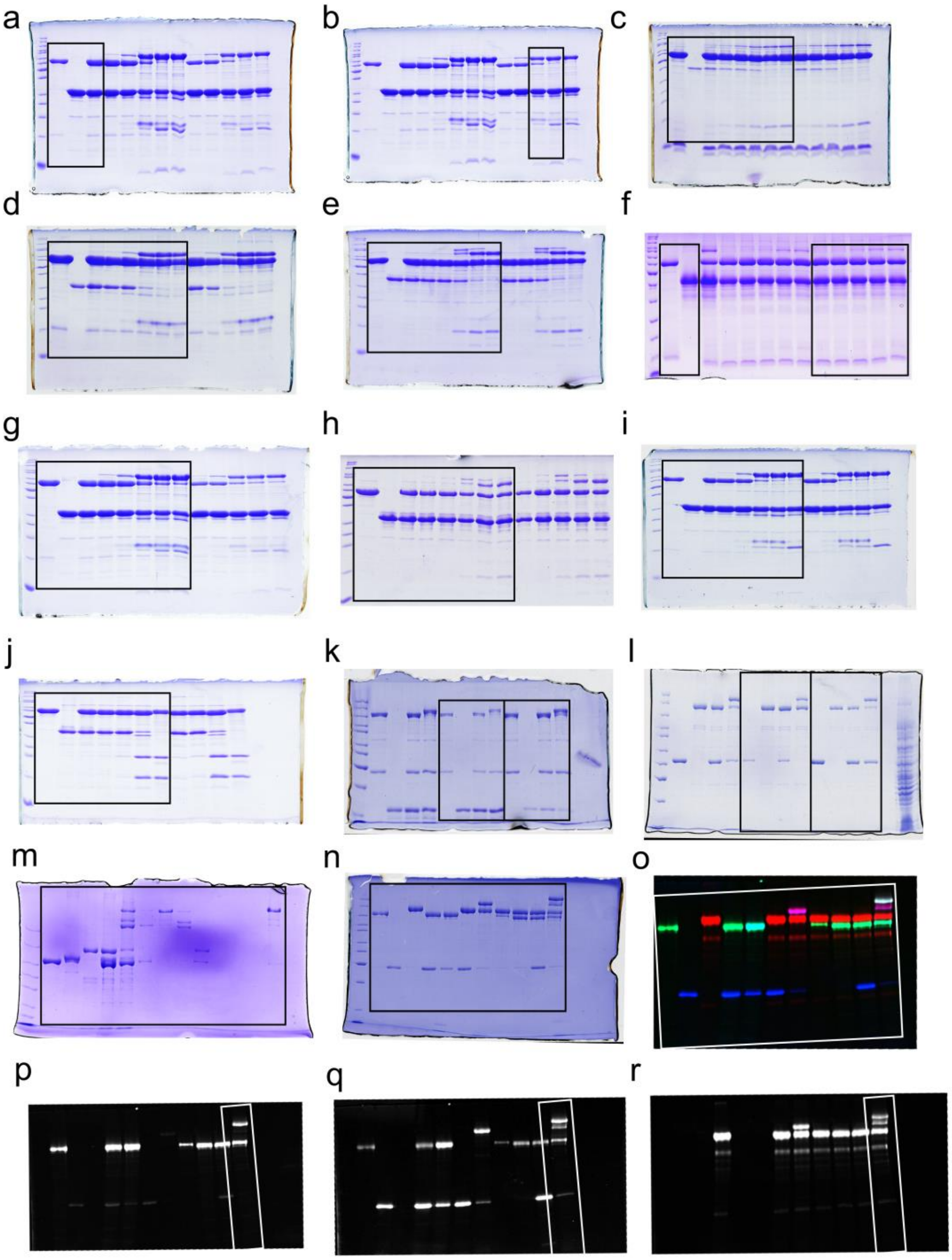

Figure S13 Unprocessed SDS-PAGE images of the figures shown in the supplementary information. The black frame indicates the section used for the figures (a) Figure S5a (left panel), (b) Figure S5b (right panel) (c) Figure S6b (left panel), (d) Figure S6b (middle panel), (e) Figure S6b (right panel), (f) Figure S7b (left panel), (g) Figure S7b (middle panel), (h) Figure S7b (right panel), (i) Figure S7d, (j) Figure S8b, (k) Figure S9a, (l) Figure S9b, (m) Figure S10, (n) Figure S11b, (o) Figure S11c, (p) Figure S11d (AlexaFluor555 channel), (q) Figure S11d (fluorescein channel), (r) Figure S11d (AlexaFluor647 channel). PageRuler™ unstained protein ladder (Thermo Scientific #26614) was used as marker.

Supplementary References

[1] S. Pietrokovski, *Protein Sci*, **1994**, *3*, 2340-2350.
[2] S. Pietrokovski, *Protein Sci*, **1998**, *7*, 64-71.
[3] Z. Charlop-Powers, J. J. Banik, J. G. Owen, J. W. Craig, S. F. Brady, *ACS Chem Biol*, **2013**, *8*, 138-143.
[4] C. Humberg, Z. Yilmaz, K. Fitzian, W. Dörner, D. Kümmel, H. D. Mootz, *Nat Commun*, **2025**, *16*, 2723.
[5] A. C. Tsolis, N. C. Papandreou, V. A. Iconomidou, S. J. Hamodrakas, *PLoS One*, **2013**, *8*, e54175.
[6] J. Abramson, J. Adler, J. Dunger, R. Evans, T. Green, A. Pritzel, O. Ronneberger, L. Willmore, A. J. Ballard, J. Bambrick, S. W. Bodenstein, D. A. Evans, C. C. Hung, M. O'Neill, D. Reiman, K. Tunyasuvunakool, Z. Wu, A. Zemgulyte, E. Arvaniti, C. Beattie, O. Bertolli, A. Bridgland, A. Cherepanov, M. Congreve, A. I. Cowen-Rivers, A. Cowie, M. Figurnov, F. B. Fuchs, H. Gladman, R. Jain, Y. A. Khan, C. M. R. Low, K. Perlin, A. Potapenko, P. Savy, S. Singh, A. Stecula, A. Thillaisundaram, C. Tong, S. Yakneen, E. D. Zhong, M. Zielinski, A. Zidek, V. Bapst, P. Kohli, M. Jaderberg, D. Hassabis, J. M. Jumper, *Nature*, **2024**, *630*, 493-500.
[7] R. C. Edgar, *BMC Bioinformatics*, **2004**, *5*, 113.
[8] M. Bhagawati, T. M. E. Terhorst, F. Füsser, S. Hoffmann, T. Pasch, S. Pietrokovski, H. D. Mootz, *Proc Natl Acad Sci U S A*, **2019**, *116*, 22164-22172.
[9] J. Rüschenbaum, W. Steinchen, F. Mayerthaler, A. L. Feldberg, H. D. Mootz, *Angew Chem Int Ed Engl*, **2022**, *61*, e202212994.
[10] T. M. Hall, J. A. Porter, K. E. Young, E. V. Koonin, P. A. Beachy, D. J. Leahy, *Cell*, **1997**, *91*, 85-97.
[11] T. Kurpiers, H. D. Mootz, *Angew Chem Int Ed Engl*, **2007**, *46*, 5234-5237.
[12] T. Kurpiers, H. D. Mootz, *Chembiochem*, **2008**, *9*, 2317-2325.
[13] T. Pasch, A. Schröder, S. Kattelmann, M. Eisenstein, S. Pietrokovski, D. Kümmel, H. D. Mootz, *Chem Sci*, **2023**, *14*, 5204-5213.
[14] N. H. Shah, E. Eryilmaz, D. Cowburn, T. W. Muir, *J Am Chem Soc*, **2013**, *135*, 18673-18681.
